# Case studies of clinical hemodialysis membranes: influences of membrane morphology and biocompatibility on uremic blood-membrane interactions and inflammatory biomarkers

**DOI:** 10.1038/s41598-020-71755-8

**Published:** 2020-09-09

**Authors:** Heloisa Westphalen, Shaghayegh Saadati, Ubong Eduok, Amira Abdelrasoul, Ahmed Shoker, Phillip Choi, Huu Doan, Farhad Ein-Mozaffari

**Affiliations:** 1 Department of Chemical and Biological Engineering, University of Saskatchewan, 57 Campus Drive, Saskatoon, SK S7N 5A9 Canada; 2 Division of Biomedical Engineering, University of Saskatchewan, 57 Campus Drive, Saskatoon, SK S7N 5A9 Canada; 3 Nephrology Division, College of Medicine, University of Saskatchewan, 107 Wiggins Rd, Saskatoon, SK S7N 5E5 Canada; 4 Saskatchewan Transplant Program, St. Paul’s Hospital, 1702 20th Street West, Saskatoon, SK S7M 0Z9 Canada; 5 Department of Chemical and Materials Engineering, University of Alberta, 243 Donadeo Innovation Centre for Engineering, Edmonton, AB T6G 2H5 Canada; 6 Department of Chemical Engineering, Ryerson University, 350 Victoria Street, Toronto, ON M5B 2K3 Canada

**Keywords:** Biochemistry, Computational biology and bioinformatics, Biomarkers, Health care, Chemistry, Engineering, Materials science

## Abstract

End stage renal disease (ESRD) patients depend on hemodialysis (HD) as a life-sustaining treatment, but HD membrane properties play a critical role in blood activation during HD and can lead to severe patient outcomes. This study reports on a series of investigations on the common clinical HD membranes available in Canadian hospitals to explore the key reasons behind their susceptibility to blood activation and unstable cytokine. Clinical HD membranes composed of cellulose triacetate (CTA) and polyvinylpyrrolidone: polyarylethersulfone (PAES: PVP) were thoroughly characterized in terms of morphology and chemical composition. Membrane-surface interactions with uremic blood samples after HD treatment were probed using Fourier Transform Infra-Red (FTIR) and Raman spectroscopic techniques in order to understand changes in chemistry on membrane fibers. In addition, as part of this innovative study, we utilized Molecular Modeling Docking to examine the interactions of human blood proteins and membrane models to gain an in-depth understanding of functional group types responsible for perceived interactions*. In-vitro* adsorption of fibrinogen on different clinical HD membranes was compared at similar clinical operating conditions. Samples were collected from dialysis patients to ascertain the extent of inflammatory biomarkers released, before, during (30 and 90 min) and after dialysis (4 h). Collected blood samples were analyzed using Luminex assays for the inflammatory biomarkers of Serpin/Antithrombin-III, Properdin, C5a, 1L-1α, 1L-1β, TNF-α, IL6, and vWF. We have likewise incubated uremic blood in vitro with the two membrane materials to determine the impact that membrane materials pose in favor of activation away from the hydrodynamics influences. The results of our morphological, chemical, spectroscopic, and in vitro incubation analyses indicate that CTA membranes have a smoother surface and higher biocompatibility than PAES: PVP membranes, however, it has smaller pore size distribution, which results in poor clearance of a broad spectrum of uremic toxins. However, the rougher surface and greater hydrophilicity of PAES: PVP membranes increases red blood cell rupture at the membrane surface, which promotes protein adsorption and biochemical cascade reactions. Molecular docking studies indicate sulfone functional groups play an important role in the adsorption of proteins and receptors. PAES: PVP membranes result in slower but greater adsorption of fibrinogen, but are more likely to experience reversible and irreversible fouling as well as backfiltration. Our major finding is that a single dialysis session, even with a more biocompatible membrane such as CTA, increases the levels of complement and inflammation factors, but to a milder extent than dialysis with a PAES membrane.

## Introduction

End stage renal disease (ESRD) affects approximately 10% of the world’s population. A healthy kidney effectively eliminates uremic protein wastes, cleans the blood, and regulates the body metabolism through a combination of glomerular filtration function, reabsorption processes, and catabolism within proximal tubule cells^1^. However, when kidneys become so damaged that they cannot maintain physiological functioning, they either must be replaced (i.e., organ transplantation) or dialysis is prescribed. Due to the cost associated with kidney transplants, the shortage of organs, and the risks associated with long-term peritoneal dialysis, ESRD patients normally opt for hemodialysis (HD)^2–4^.

HD therapy is a life-sustaining treatment; however, this membrane-based therapy is also plagued with some acute side effects and associated with chronic health conditions and life-threatening disorders. Gross levels of renal byproducts within the serum increase in the body when renal function fails. Some of these protein byproducts are present in remarkably high concentrations in patients with chronic renal failure, especially those undergoing long-term HD. These uremic proteins can be classified according to their molecular weight as either small water-soluble compounds, mid-molecular weight compounds, or protein-bound toxins^1^. High concentrations of these proteins in HD patients may result in complicated health issues (e.g., chronic ischaemic heart disease); most cause physiological oxidative stresses and chronic kidney diseases that subsequently lead to death^2^. In some patients, increases in hematocrit lead to unstable predialysis creatinine and uric acid concentrations^3,4^.

Due to bioincompatibility, the interactions between most HD membranes and the blood can lead to adverse inflammatory reactions. Specifically, the exposure of blood to HD membranes triggers complement, coagulation, and thrombosis activations^5^. The coagulation cascade is activated by contact between the blood and exogenous materials during extracorporeal circulation. In this case, an acute inflammation begins quickly, becoming severe within a short period of time, and with symptoms lasting for multiple days. Triggering of the coagulation cascade is traditionally inhibited by the administration of heparin. While the medical consequences of cell activations are not fully explained, various pro-inflammatory mediators and cytotoxic materials are known to increase and thus cause chronic inflammation, which in turn involves the cardiovascular system^6^. The coagulation and complement systems take on essential functions throughout the inflammatory response. The complement system, including monocytes, macrophages, and others, under the control of T helper cells, plays a critical role when it comes to immunoprotection^7^. The coagulation cascade, on the other hand, enhances thrombin generation through the intrinsic pathway where the cascade’s aim is to ensure hemostatic maintenance^8^. As per mechanistic understanding, these cascades are initiated near foreign surfaces. The proteins of the complement and hemostasis systems are primarily synthesized during contact with superficial areas on the endothelium, circulating entities such as platelets, pathogens, and leukocytes on various artificial surfaces. In the activation of both cascades, factor X initiates prothrombin using a series of factors. Active thrombin can then catalyze the polymerization of fibrin and succeeding fibrin polymers then lead to clots, together with activated platelets^9^.

The physical and chemical properties of HD membranes readily contribute to the inflammatory response during complement activations, and this remains a key aspect in understanding their biological compatibility^10^. According to Olafiranye et al*.*^10^, these interactions are also responsible for platelet activation and membrane-surface adhesion^11^. However, membrane chemistry may be the major difference between membranes from natural vs. synthetic sources. Complete or partial replacement of hydroxyl chemical groups in modified cellulose membranes may affect their biocompatibility, as acetylation of these hydroxyl groups remarkably improved membrane biocompatibility, leading to reduced thrombus formation and platelet adhesion^12^.

A number of studies have considered some aspects of protein adsorption with respect to the polymers used in HD membranes, including the influence of membrane morphology on protein adsorption as well as general HD performance. Some researchers have reported the use of semipermeable polymer membrane adsorbents in peritoneal dialysis systems^13,14^, functionalized dialysis membrane materials for dialysis applications^15,16^, and the impacts of dialysis membranes on outcomes in acute renal failures^17^. Recently, Bensaadi et al.^18^ studied the effects of molecular weight of polyvinylpyrrolidone (PVP) and polyethylene glycol (PEG) additives within cellulose triacetate (CTA) hybrid membranes for dialysis. In this study, the presence of PVP contributed to enhanced membrane morphology and transport performance depending on the molecular weight of the PVP. Irfan et al.^19^ also investigated the HD performance of PVP/carboxylic-multiwall nanotube nanocomposite blended with polyethersulfone (PES) membrane. These authors report a nearly 30% improvement of hydrophilic behavior for this membrane with a very reduced leaching ratio of inorganic additives (approximately 1.89%) at an improved water flux. This membrane also showed improvement against protein fouling (bovine serum albumin and lysozyme) compared to the unmodified membrane. Our research group also investigated the chemical groups responsible for enhancing dialysis membrane hemocompatibility^20,21^. The extent of protein adsorption on the polysulfone membrane surface hydrophilized with PVP has also been investigated with fibrinogen and human serum albumin (HSA)^21^. HSA adsorption was inhibited compared to fibrinogen due to the membrane’s hydrophilic surface. Other authors recently studied the potential and charge density distributions of hollow-fiber dialysis membranes, including CTA^22^; bromelain immobilization on CTA membrane derived from sugarcane bagasse^23^; phosphine oxide incorporation within CTA membranes for p-cresol adsorption in serum^24^; and the relationship between peritoneal dialysis durations in end-stage renal patients^25^. The application of membranes to kidney dialysis is an ongoing materials science research field and its importance is rapidly growing. However, improvement of membrane hemocompatibility calls for in-depth investigations of several critical factors. HD brings with it a wide range of complications and side effects that can have a negative impact on patient quality of life and lead to unacceptably high mortality and morbidity rates^5^. Nonetheless, even with extensive research in this field, full understanding of the effects of blood-membrane interactions are incomplete as they relate to biocompatibility.

Therefore, this study aimed to improve understanding of the performance of common clinical membranes used in kidney dialysis in Canadian hospitals, specifically those composed of cellulose triacetate (CTA) and polyvinylpyrrolidone: polyarylethersulfone (PVP: PAES). The objectives of the study were to: (i) investigate the effects of critical membrane characteristics (e.g., morphology, hydrophilicity, and chemistry) on induced complement activation and inflammation; (ii) comprehensively examine the interactions between membrane materials and human serum after dialysis treatment to uncover the influence of membrane fiber chemistry; (iii) critically assess how these factors influence the inflammatory biomarkers released during HD at different dialysis treatment times; (iv) use molecular modeling of docking to access the interaction of human blood proteins with membrane models to gain an in-depth understanding of the functional groups responsible for the interactions as well as the protein adsorption capacities of both membranes; and (v) examine the effects of membrane chemistry on the release of inflammatory biomarkers in patients’ uremic blood.

## Experimental

### Materials

This study used actual clinical membrane modules utilized in Canadian hospitals, comprised of either CTA (Exeltra 210 dialyzer) or a blended PAES:PVP polymer (also referred to herein as simply PAES) (REVACLEAR 400 dialyzer). These medical-grade membranes were supplied by St. Paul’s Hospital, Saskatoon, Canada, and are recognized in the medical field for their optimal filtration flux, solute removal, and hemocompatibility. New and used dialyzers were utilized for comprehensively investigating membrane characteristics before and after HD treatment. In addition, the membrane-surface interactions after HD treatment were examined using used dialyzers. Glutaraldehyde (95%), osmium tetroxide (95%), and sodium cacodylate (95%) were purchased from Sigma Aldrich and used as received without further purification.

### Advanced surface visualization of clinical membrane fibers via imaging techniques

Atomic force microscopy (AFM) images were collected on the fibers of both types of clinical membranes using a Model 4,500 AFM instrument (Keysight Technologies, Chandler, AZ) operating in intermittent contact mode. AFM measurements were conducted with the aid of a conical silicon cantilever (Applied NanoScience, Tempe, AZ) with a 50 N m^−1^ force constant, 170 kHz resonant frequency, and less than 10 nm curvature radius. All measurements were taken with the ratio of the set-point oscillation amplitude to free air oscillation amplitude at 0.90, and resonant amplitude in the range from 11 to 17 V. In addition, all measurements were performed at 25 °C with the instrument mounted in a vibration isolation system with a scan rate of 1.0 Hz (512 pixels per line) for all images. The CTA and PAES membrane fibers were mounted on glass substrates using double-sided tape for AFM measurements, which were conducted on 3–4 different spots on the inner/outer surfaces. The PAES fiber, in particular, was cut to expose its internal structures. Briefly, an incision was made longitudinally along this membrane fiber, which was opened and secured on the glass substrate. The CTA fiber was cut longitudinally, but could not be opened; therefore, no AFM measurement was completed for the internal CTA fiber. Values of surface roughness (average R_a_, and root mean square *R*_RMS_) from each surface analyzed were correlated with the tendency toward hemolysis and other complications related to biocompatibility.

Membrane morphologies before and after dialysis were analyzed using a Hitachi SU8010 scanning electron microscope (SEM) at the Western College of Veterinary Medicine (WCVM) Imaging Centre, with images collected at 3 kV acceleration voltage. Care was taken to avoid creating burrs in the Au-precoated (10-nm; Quorum Q150T ES) samples with excess electron volts while collecting images at high magnifications. These coated layers allowed for visualization without the external influence of any adsorbed contaminant due to the presence of conductive surfaces at this acceleration voltage. SEM imaging was performed on both clean and blood-contacted CTA and PAES membrane samples after HD therapy. Analyses were carried out after cell/tissue fixation, using a fixation medium composed of 2% glutaraldehyde or 1% osmium tetroxide in 0.1 M sodium cacodylate (pH 7.2). After 1 h fixation, the membrane fibers were washed twice for 10 min (each wash) in a cacodylate buffer and finally rinsed in distilled water. The blood-contacted samples were also cut into cross-sections to observe the blood cells that adhered within the internal walls of the membrane. The membrane tubes were exposed so as to collect images of their internal contents while mounted on Al stubs with a piece of double-sided carbon sticky tape.

Impacted and fouled membrane filters after ultrafiltration were also analyzed by transmission electron microscopy (TEM; Hitachi HT 7700) to reveal any changes in membrane microstructures. Similar to SEM analyses, in-depth surface examinations were accompanied by analysis of the extent of protein fouling. Blood-contacted membranes were coated with 1% ultrapure low melting point agarose for fouling stabilization after cell/tissue fixation. Samples were transitioned into BEEM capsules with fresh LR White embedding medium and held overnight at 60 °C before sectioning on a Leica ultra-cut ultra-microtome to a thickness of 90 nm. Sections were then observed on a 200-mesh copper grid. In TEM imaging, higher electron density results in darker regions and vice versa; however, samples were osmicated so density changes may be due to absorption of the osmium.

### Advanced chemical and spectroscopic analyses of clinical membrane fibers

Raman spectroscopy measurements were carried out on a Renishaw InVia Reflex Raman microscope using an Ar + laser (Modulaser StellarPro-50) operating at 514.5 nm and an 1,800 line mm-^−1^ grating. The microscope focused onto the sample using Leica 50X NPLAN (NA = 0.75) or 100X NPLAN (NA = 0.90) objectives, and the backscattered Raman signals were collected with a Peltier cooled CCD detector. Measurements were collected using extended scan and a 10 s detector time. The laser power was 2.7 mW (50X objective) or 1.4 mW (100X objective) measured at the sample. The instrument was calibrated using an internal Si(110) sample, which was measured at 520 cm^−1^. Raman maps were generated using the instrument in Streamline mode using the 514.5 nm and 1,800 line mm^−1^ grating. The detector time was set at 10 s and each pixel was 1.2 × 1.2 μm for the 50X NPLAN objective and 0.6 × 0.6 μm for the 100X NPLAN objective. Raman images were processed using Renishaw Wire V3.4 software. Preparation of the samples of both membrane fibers was conducted by WCVM Imaging Centre staff. Polymer fibers were embedded in OCT (water-soluble blend of glycols and resins) and cryo-microtomed into 10- to 20-μm sections. These membrane sections were mounted on Au-coated Si wafers and gently rinsed with water to remove any residual OCT prior to Raman imaging measurements.

To complement the chemical analyses, X-ray photoelectron spectroscopy (XPS) analyses of the membranes were further conducted with the aid of a Kratos AXIS Supra X-ray Photoelectron Spectrometer (Kratos, UK) with a monochromated Al K $\alpha$ X-ray source. The spectral data were collected and analyzed from samples at less than a 100-μm spot size and at a 90° emission angle. The spectra (between 0 and 1,200 eV) presented within this work are from wide scans collected with the aid of CasaXPS software.

Attenuated total reflectance-Fourier transform infrared (ATR-FTIR) spectra of both membrane fibers were collected using a Renishaw-inVia Raman Microscope (Renishaw, UK) in transmittance mode. The FT Raman spectra collected within this work are also accompanied by surface mapping. Spectral results were collected from clean and blood-contacted membrane samples (both CTA and PAES) to investigate membrane/blood interactions.

The zeta potential and Brunauer–Emmett–Teller (BET) surface area of the membrane fibers were analyzed using a zeta potential analyzer and BET analyzer, respectively, to investigate the influence of particle size and surface charge on membrane filtration efficiency. Surface charge measurements for both HD membranes were conducted using a zeta potential analyzer (Zetasizer-Nano Series, Malvern Instruments Ltd., UK, ± 0.01 mV). Zeta potential measurements were made at pH 7 with 2 mM KCl solution. The molecular weight cut-off for the membrane samples was assessed using a BET ASAP 2020 system (Micromeritics, Georgia, USA). Before BET measurements, samples were degassed at 50 °C for 3 h to remove any moisture and inert nitrogen gas was incorporated during the experiments.

### Molecular docking theoretical computation

1-Phenoxy 4-(phenylsulfonyl) benzene and (2S,3S,5S,6S)-6-(acetoxymethyl)-2-(((2S,4S,5S)-2,3-diacetoxy-5-(acetoxymethyl)-6-methoxytetrahydro-2H-pyran-4-yl)oxy)-5 methoxytetrahydro-2H-pyran-3,4-diyl diacetate were selected as monomer models (ligands) for the PAES and CTA membranes, respectively. The structures were drawn in Chemdraw software and the Chemdraw format of the ligand then converted to pdb format (Fig. S1a,b). Energy minimization was performed for the structure using Chem3D Ultra (Version 8.0) software (Fig. S1c,d).

Docking studies were carried out using AutoDock software version 4.0 to determine favorable structural characteristics for protein–ligand interactions. The three-dimensional X-ray structures of fibrinogen (PDB code: 2VDM), albumin (PDB code: 2BXD), transferrin (PDB code: 4X1B), P2Y12 (PDB code: 4NTJ), heat shock protein (HSP; PDB code: 5MKR), and hemoglobin (PDB: 2HHB) were chosen as templates for the modeling study. Water molecules were removed from the protein, Kollman charges were added, nonpolar hydrogens were merged, and finally AutoDock 4 atom types assigned to achieve the PDBQT format of the protein structure. The model was created and minimized using HyperChem 8.0 and then converted to PDBQT file format with AutoDock tools. The active site was defined as a grid box around the crystallographic ligand in 20 × 20 × 20 dimensions. The Lamarckian genetic search algorithm was employed, and the docking run was set to 50. Protein residues with atoms greater than 7.0 Å from the docking box were removed for efficiency. Types of interactions of the docked protein with the model were analyzed after the end of molecular docking by measuring the intermolecular energy of the model-protein assembly.

### Fibrinogen adsorption

The extent of protein fouling and adsorption on the polymer membrane fibers were determined by filtering a model protein (fibrinogen, FB) from a simulated blood solution made of FB from human plasma (Sigma-Aldrich), saline (0.9% NaCl Injection USP, Baxter), and phosphate buffered saline (PBS; 1.0 M, pH 7.4 at 25 °C, Sigma Aldrich). Dialysate was created from the following reagents (Sigma Aldrich) dissolved in distilled water: sodium chloride (NaCl, ≥ 99.0%), calcium chloride (CaCl_2_, ≥ 93.0%), potassium chloride (KCl, ≥ 99.0%), sodium bicarbonate (NaHCO_3_, ≥ 99.7%), magnesium chloride (MgCl_2_, ≥ 98.0%), sodium acetate (CH_3_COONa, ≥ 99.0%), and glucose (anhydrous, 96%). The detailed composition of dialysate solution is shown in Table S1. Five samples of known FB concentration (0.06, 0.12, 0.25, 0.50, 1.0 and 2.0 mg/mL) were carefully prepared at room temperature (22 °C) from 0.9% NaCl saline solution (Baxter) and FB from human plasma. The pH of the solution was adjusted to 7.2 using PBS. The samples were analyzed using a UV–Vis spectrometer (Flame, Ocean Optics). The UV/Vis spectrum for the five known concentrations of FB as well as the calibration curve for FB concentration are presented in Figs. S2 and S3.

A simulated HD procedure was carried out using the PAES and CTA membrane clinical modules. The experiments were conducted in triplicate. The 2.0 mg/mL FB solution described above was used to simulate the composition of HD patient blood. The solution was maintained at 37 °C with constant gentle stirring. The FB solution and the dialysate solution were pumped through the dialyzer in countercurrent mode. During the operation, the flow rate of the FB solution (Qb) and dialysate solution (Qd) were kept constant, while the pressure was monitored in all inlets and outlets. The blood flow rate of 300 mL/min and dialysate flow rate of 500 mL/min aimed to match common clinical operating conditions. The adsorption experiment was run was until equilibrium was achieved. Small samples were collected from the reservoir containing the FB solution every minute and the concentration of FB in each sample was analyzed using UV/Vis at room temperature.

### Inflammation/complement activation test

Clinical tests were conducted to ascertain the extent of inflammatory biomarkers released before and during HD sessions. A cohort of eight HD patients using CTA and PAES dialyzers; and two healthy controls from St. Paul’s Hospital dialysis centre were recruited. Blood samples were collected from HD patients using either CTA or PAES dialyzers following ethical approval of the study. Samples were collected before dialysis, at 30 min, 90 min, and then at the end of a 4-h dialysis session (240 min). Blood and dialysate flow rates were 300 and 500 mL/min, respectively. All blood specimens were collected using standard Vacutainer-type blood collection tubes and processed to serum by the vendor. Sample aliquots were stored frozen at − 80 °C before analysis. Inclusion criteria were classification as either normal (control) or having kidney disease (HD patients), male or female, and under 60 years of age; samples were randomized prior to analysis across three 96-well plates for the presence of serpin/antithrombin-III, properdin, complement component 5a (C5a), interleukin (IL)-1α, IL-1β, tumor necrosis factor (TNF)-α, IL-6, and the Von Willebrand factor (vWF), as described in detail in the Supporting Information. All samples were diluted with calibrator diluent based on manufacturer protocols. The fluorescence responses and concentrations of analytes were obtained using a Bio-Plex Pro Human Inflammation Panel 37-Plex assay kit with magnetic beads and analyzed with a Luminex 200 system and the accompanying Bio-Plex Manager Software 6.1 (Bio-Rad, Hercules, California, USA)^26^. Concentrations were determined from standard curves generated from each kit’s standard using the Bio-Plex Software Manager weighted 5PL curve fitting procedure. To maximize the number of concentration values available for analysis, we included the Bio-Plex extrapolated values. Therefore, the definition of out-of-range here, and unless otherwise stated, refers to concentration values that cannot be obtained from the 5PL logistic curve. Statistical analysis was performed using the individual groups t-test. Data were expressed as mean values ± SE. P value of less than 0.05 was considered as indicating a significant difference. All samples and controls were measured in triplicate, with mean, standard deviation, and p values reported.

### In vitro incubation of membranes in uremic blood

PAES and CTA membranes were incubated in ~ 100 µL serum samples from patients normally treated with either CTA or PAES dialyzers (n = 3), to eliminate the hydrodynamic influence, as well as the side effects of the shear stress and the ruptured blood cell due to higher roughness. In addition to investigating how the membrane material of specific dialyzer can trigger more blood activations of patients normally treated with the opposite membrane. Incubations were conducted at 37 °C in separate Eppendorf tubes. After 30 min, the membrane samples were then removed and 1 µL aliquots of the serum were prepared and subjected to Luminex assays. All samples and controls were measured in triplicate, with mean, standard deviation, and p values reported. Data were expressed as mean values ± SE.

### Research ethical principles of human experimentation

Dr. Amira Abdelrasoul, the principal investigator of the project, obtained University of Saskatchewan Research Ethics Approval as well as Saskatchewan Health Authority Operational Approval to conduct the research in Saskatchewan Health Authority, in Canada. All the experimental protocol for involving humans was conducted according to the governing law. All study participants from St. Paul’s Hospital signed a written informed consent, approved by the University of Saskatchewan Biomedical Research Ethics Board.

## Results and discussion

### Morphological and chemical characterizations of clinical membrane fibers

By definition, a typical HD membrane removes toxins and, at the same time, prevents vital proteins from being lost due to its active separation and nanofibrous support layers. Its uppermost porous multilayers are designed for the desired surface need (e.g., hydrophilicity) to enable permeability and selectivity. The second layer underneath is composed of nanofibers with interconnecting nanoporous structures that also offer pathways for removing excess water and protein-based toxins (e.g., metabolites)^27^. Membrane layers are able to withstand pressures of the treatment processes while some degree of hydrophilicity may be required to ensure improved blood compatibility and reduced protein adsorption. In addition, surface roughness plays an important role in the interaction between blood components and the membrane surface; increased roughness leads to an increase in the shear stress experienced by the blood components flowing near the membrane surface, which can lead to hemolysis or rupture of red blood cells (RBCs). This phenomenon can lead to the manifestation of many clinical symptoms ranging from headache, back pain, and hypertension to even death^28^. Additionally, RBC rupture can enhance protein adsorption and platelet adhesion, leading to coagulation pathways and thrombus formation.

**Figure 1:**
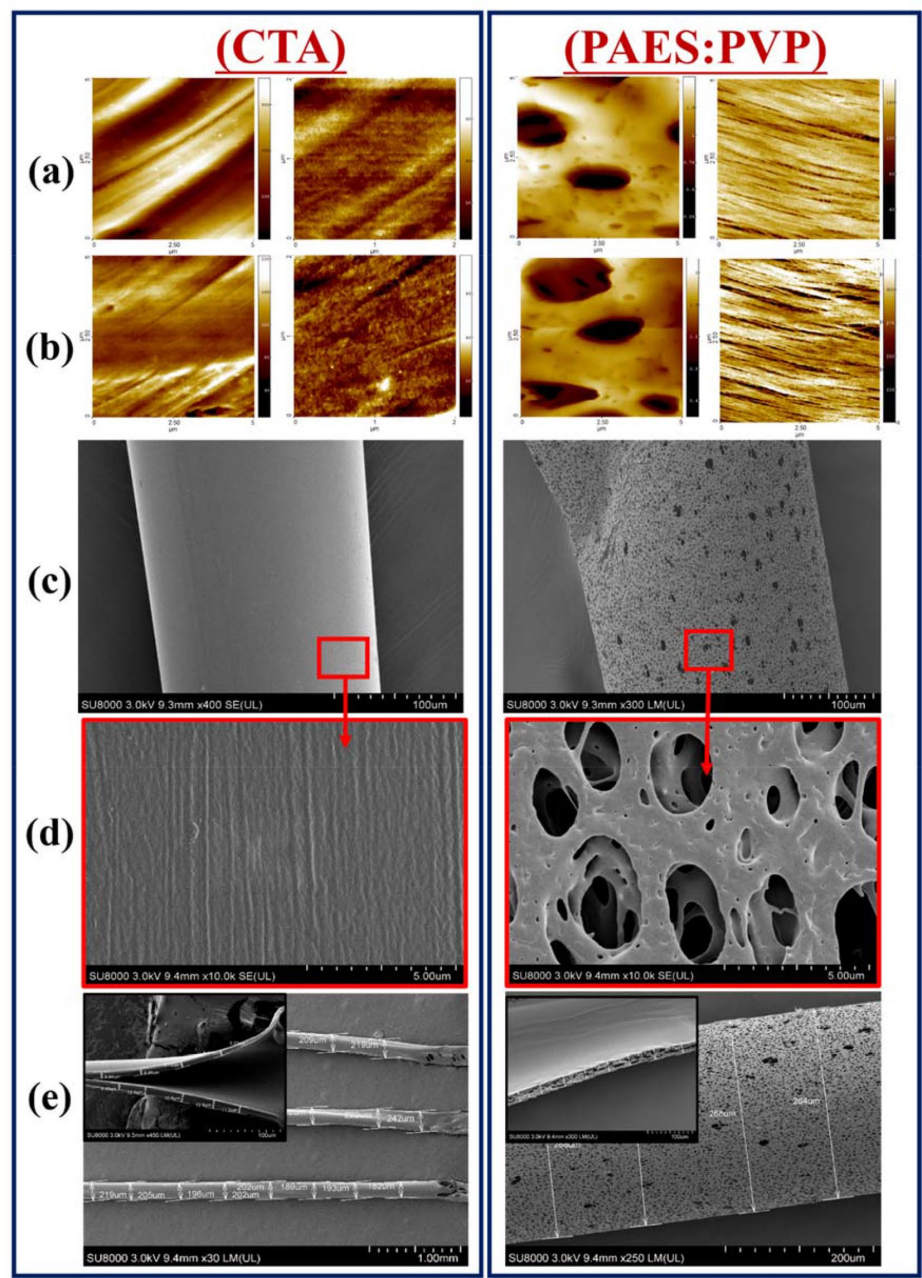
AFM micrographs of pristine CTA and PAES HD membrane fibers collected for two different spots in close proximity (**a** and **b**, first second rows); micrographs of the inner and outer surfaces of the polymer membrane fibers are placed in the first and second columns, respectively. SEM micrographs of CTA and PAES HD membrane fiber surfaces (**c**), magnified surface images thereof (**d**), and whole microtubes (**e**).

Figure 1 depicts AFM micrographs of CTA and PAES HD membrane fibers. The CTA images show prominent fibrous strains indicated as lined strokes, whereas the internal and external features of the PAES fibers are distinctly porous and stretched/lined. The porous side could make up a sectioned separation layer with an active pathway for diffusive transport of solutes. Corresponding magnitudes of average roughness (R_a_) and root mean square roughness (R_RMS_) are summarized in Table S2.

The surface roughness values of the PAES membrane, both the inner and outer layers (10.4 ± 4.0, and 15.3 ± 5.9 nm, respectively) were greater than corresponding values for the CTA membrane (5.4 ± 1.9 and 7.5 ± 3.0 nm, respectively). This means the CTA membrane had a smoother surface and therefore is expected to result in less hemolysis compared to the PAES membrane. Consequently, the CTA membrane should have reduced protein adsorption and hence facilitate less blood activation (see Sect. 3.5). Patients undergoing dialysis using PAES membranes or other HD dialyzers with rough membrane surfaces may experience more symptoms associated with hemolysis and other complications due to protein adsorption.

SEM surface images of whole membrane microtubes are presented in Fig. 1c–e and illustrate membrane thickness and pore formation. Membrane morphology contributes to filtration efficiency^29–32^. The PAES membrane contains more interconnected porous structures within its fibers (Fig. 1c–e) compared to the CTA membrane (Fig. 1a,b). Similar surface features have been reported in other studies^18,29^. CTA fibers show stretched, lined, and rather flat structures with sharp pore distributions on the surface. These fibers have a more uniform surface and appear to be denser, with no apparent porosity and homogeneous lined microstructures. The CTA and PAES microfibers have average diameters of 185 and 266 μm, respectively. The microfibrous layers of the CTA membrane microtube substrate have a more compact thickness (8.9–12.9 μm) while those of the hybrid PAES fibers show no distinct thickness changes within the measured length (21.7–24.0 μm). Therefore, CTA membranes may have a higher solute permeability that can remove more β2-M by diffusion relative to PAES membranes. The diffusive efficiency of CTA membranes is very high because the fibers are thin and have a unique structure (Fig. 1), causing the flow distribution of the dialysate to be uniform. The larger fiber thickness of the PAES membrane could also be linked to the uniform PVP polymer layer of the asymmetrical PAES support structures^33^. On the other hand, the CTA membrane did not show a broad pore size distribution, which could contribute to poor clearance of a broad spectrum of uremic toxins relative to the PAES membrane.

It was essential to also explore the membrane pore sizes and net porosity because the membrane is the core element of the HD process. During HD, toxic metabolites and excess water from the blood are eliminated by diffusive and convective transport across the membrane while loss of necessary proteins is prevented due to pore size exclusion of the membrane. BET surface area and zeta potential values are summarized in Table S3. The CTA fibers exhibit a high surface area (14.47 m^2^ g^−1^) relative to PAES fibers (1.99 m^2^ g^−1^). The SEM and BET analyses show the PAES membrane had a broad pore size distribution compared to the CTA membrane. In addition, the CTA membrane is thinner and has a higher surface area and remarkably smaller pore size compared to the PAES membrane. These morphological characteristics have a notable impact on HD, especially in terms of toxin removal depending on the pore size, protein loss, and protein adsorption (see Sect. 3.4). Using CTA membranes with small pore sizes could effectively remove small toxins, but might also lead to retention of undesired middle-size molecules. On the other hand, PAES membranes (the most common in Canadian hospitals), which have larger pores than CTA membranes, can operate at high flux and promote clearance of both small and middle-size molecules. However, this can lead to depletion of physiological proteins and increase the potential for backfiltration, which can reintroduce toxins from the dialysate side back to the blood stream^34^. Pore size has an impact on the adsorptive behavior of HD membranes; specifically, membranes with different pore sizes adsorb different proteins and therefore different outcomes related to blood activations can be expected to be mediated by protein adsorption^35,36^.

The zeta potential for CTA fibers was − 34 mV and for PAES fibers was − 68 mV, and can be used to predict a membrane’s capacity for fouling due to its surface hydrophilicity. The negative zeta potentials reflect remarkable foulant deposition on impacted surfaces. The zeta potential of fibers also plays a major role in the diffusion of solutes across the membrane and charges are linked to the inherent presence of chemical functional groups within them. The CTA fibers possess non-ionizable acetate (–COCH_3_) and hydroxyl (–OH) functional groups that do not have a fixed charge^37–39^ compared to the aromatic polysulfone on the PAES fibers. Theoretically, CTA has no fixed charge, but some anions may preferentially aggregate within its surface, in turn yielding a slightly negative zeta potential^40,41^. A more hydrophilic surface is expected to lead to less protein adsorption and less deposition and fouling of blood on the membrane surface. Importantly, however, adding more hydrophilic structures (functional groups) to the polymer structures to increase the hydrophilicity has not resulted in higher blood compatibility^42^. However, when blood touches the hydrophilic polymeric structure, more water molecules would be adsorbed from blood, which may cause blood activation. With a substantial amount of protein adsorption to the membrane surface, the surface charge would be neutralized and hence the adsorptive properties would change during the HD process.

The surface chemistry of both sides of these polymer fibers was further comparatively examined using XPS. The spectra for respective sample surfaces were analyzed with the aid of the CasaXPS software as presented in Fig. S4. The spectra of each side of the each material show similar peaks with no discernable differences due to similarities in chemistry; however, notable differences were evident when comparing the CTA and PAES membranes. The spectra for the CTA membrane reveal common elements, mainly C and O, with less C content observed on the inside (64.37% C and 29.30% O) than outside (74.51% C and 21.97% O) of this membrane fiber. Except for traces of other elements related to polyarylethersulfone, the spectra of the PAES membrane also reflect C 1*s* (285 eV), O KLL (988 eV), and O 1*s* (530 eV) peaks on the inside and outside. The C and O contents of the two sides are the close, with 75.60% C and 12.29% O (inside) and 76.47% C and 13.56% O (outside). The diarylsulfone moiety in PAES also contributed to faint S 2*s* and S2p peaks at 227 and 168 eV, respectively, while the N 1*s* peak at 400 eV could be attributed to PVP. S and N are present at less than 4% on both sides of the PAES fibers. These data show the low blending ratio of PVP to PAES. Elemental contents are associated with the 100-μm spot size sampled and analyzed for each polymer fiber. Table S4 shows the percentage abundance of elements within these membrane fibers.

In-depth analyses of the fibers of both membranes before dialysis treatment were accomplished using Raman spectroscopy. Figure S5 shows optical images and Raman mappings of the surfaces (first and third rows) and cross-sections (second and fourth rows) of the CTA and PAES: PVP hollow membrane fibers. For the CTA fibers, the observed Raman images were mapped from dimensions of 80 × 43 µm (1.2 × 1.2 μm pixel size) for the surface and 36 × 54 µm (1.2 × 1.2 μm pixel size) for the cross-section at an excitation wavelength (λ_ex_) of 514.5 nm. C–H stretching vibrations, likely associated with the acetate chemical group, were mapped for defined wavelength regions between 2,910–2,980 (Fig. S5b) and 2,700–2,750 (Fig. S5c) cm^−1^. For PAES:PVP fibers, the observed Raman images were mapped from dimensions of 67 × 131 µm (1.2 × 1.2 μm pixel size) for the surface and 84 × 62 µm (0.6 × 0.6 μm pixel size) for the cross-section. The vibration band linked with the aromatic phenyl ring of sulfone chemical groups was mapped at wavelength regions presented in Fig. S5e,f; vS–O bond stretching vibration is responsible for the peak at 1,145 cm^−1^. The last columns show maps collected from ratios of a and b as well as e and f for CTA and PAES:PVP, respectively.

### Chemical changes of membranes after interactions with uremic blood (after HD treatment)

**Figure 2:**
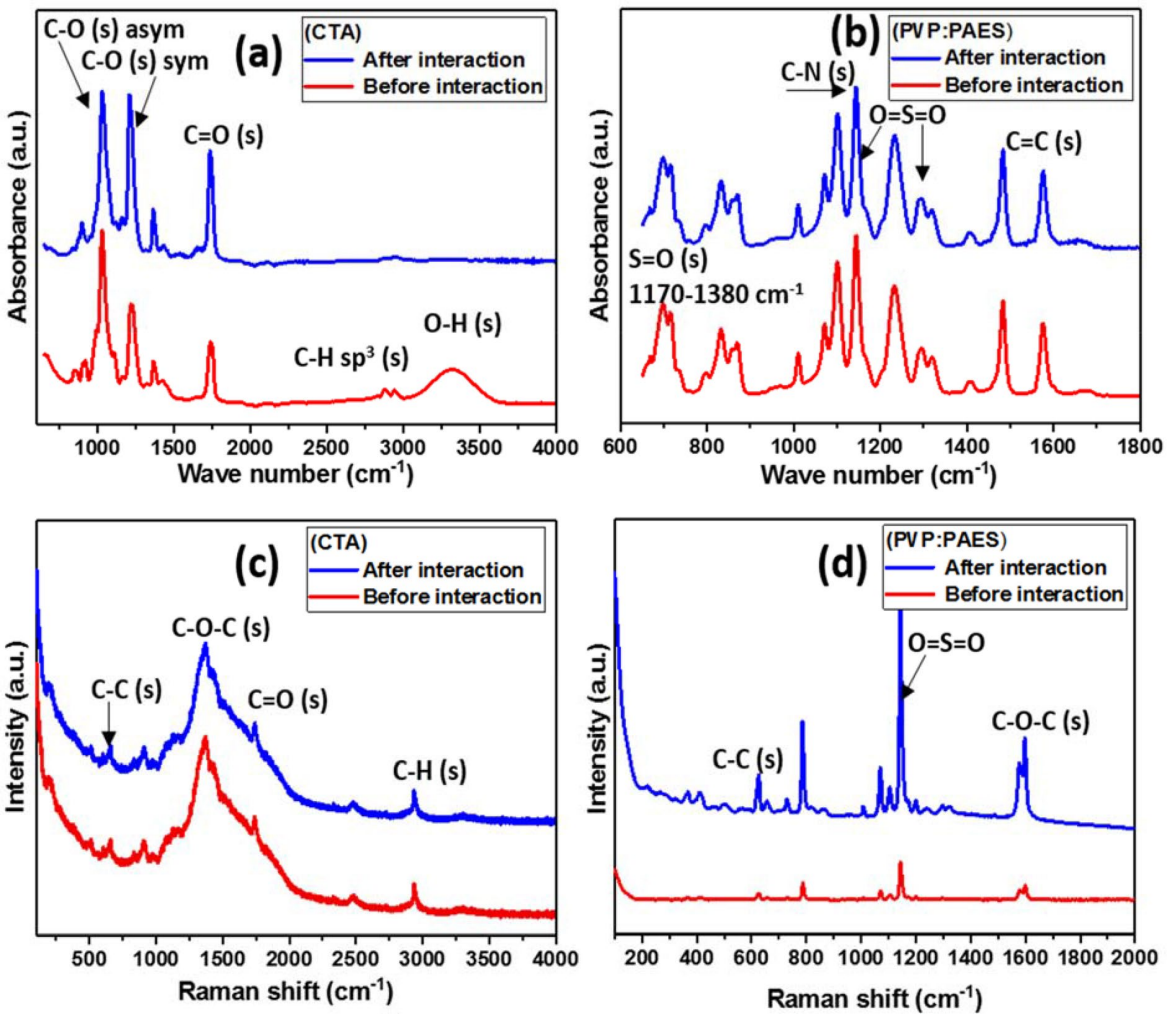
FTIR and Raman spectra of CTA (**a**, **c**) and PAES (**b**, **d**) HD membrane fibers before and after interaction with uremic blood.

Evidence of chemical changes within membrane fibers after interacting with uremic blood were investigatedutilizing used dialyzers after HD treatment. Figure 2 presents FTIR and Raman spectra of CTA (a,c) and PAES (b,d) HD membrane fibers before and after interaction with uremic blood at the end of HD treatment. The major IR spectral feature for the CTA fiber is the absorption band around 1755 cm^−1^, corresponding to the carbonyl group (stretching vibrations). Other peaks can be attributed C-O bonds (asymmetric and symmetric stretching, vibration modes, respectively) around 1,032 and 1,246 cm^−1^^29,33^. The small shoulders around 2,900 cm^−1^ are adsorption bands of C–H bonds. The spectrum of the PAES fibers shows the presence of PVP is not conspicuous; only a very weak signal intensity is evident at 3,475 cm^-1^ due to N–H bond (stretching modes) on the pyrrolidone’s cyclic amine ring. The most distinguishing structural feature in the PAES spectrum is the para-linked diarylsulfone moiety at 1,137 and 1,307 cm^−1^. The IR spectrum of the PAES fiber shows bands corresponding to C=C bonds (stretching vibration modes) at around 1,600 cm-^−1^ due to the aromatic rings (on PAES)^29^.

Raman spectra for both fibers are presented within Fig. 2c,d and mapping in Fig. S6. Raman peaks corresponding to CTA-related chemical groups (C–C, C–O–C, C=O, and C–H stretching vibrations) are assigned in Fig. 2c. The peak corresponding to C–H stretching is observed around 2,900 cm^−1^ while the one at 1,720 cm^−1^ is attributed to stretching of the carbonyl group (within functional carboxyl groups). The observed small peaks at 1,350 and 1,520 cm^−1^ are assigned to asymmetric and symmetric carboxyl stretching vibrations, respectively. However, for both sides of the CTA membrane fiber, peaks related to C–OH bond (stretching) and out-of-plane –OH bonds are not conspicuous at 1,040 and 632 cm^−1^, respectively. Absorption peaks characteristic of the symmetric and asymmetric stretching vibrations of sulfone groups on PAES are located at 1,170 and 1,336 cm^–1^. Fouling based on contact with uremic blood of real patients reveals slight chemical changes^43^. Shifts in absorption bands and alteration in peak intensities, no matter how minute, could be ascribed to the adhesion of blood aggregates. PAES demonstrated a higher propensity for fouling and revealed more chemistry changes compared to CTA. Several studies show a distinct correlation between fouling potential and heterogeneous property distribution (e.g., foulant adhesion forces)^40,44^. These adhesion forces relate to surface chemistry/charge, chemical groups, fiber morphology, and even roughness^45^.

**Figure 3:**
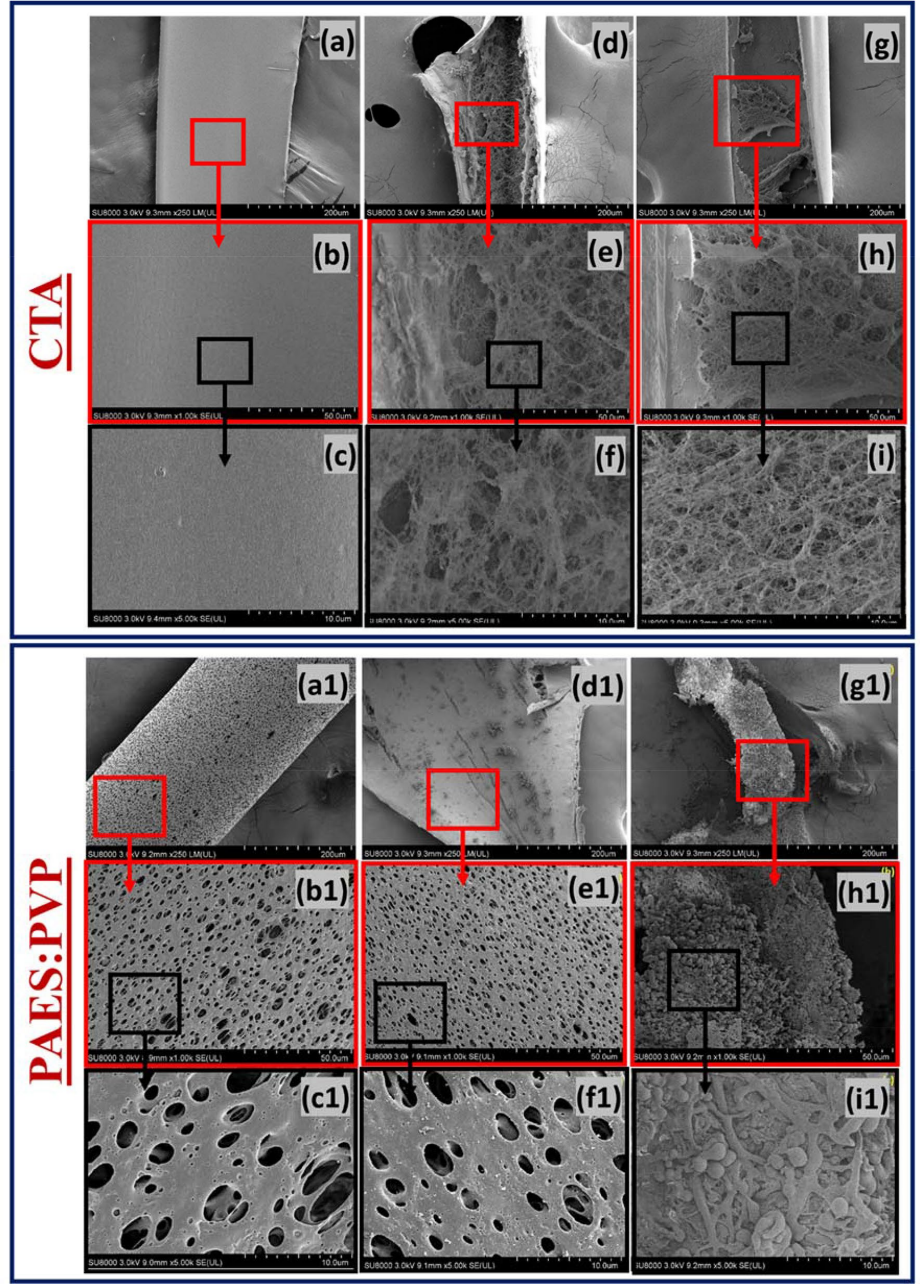
Surface SEM micrographs of CTA and PAES HD membranes before and after dialysis treatment; the first column presents the pristine membranes while the second and third columns show blood-stained images at different magnifications with evidence of pore blocking with blood/membrane aggregates inside the membrane microtubes.

Figure 3 shows surface SEM micrographs of pristine and blood-contacted CTA HD membranes. Before HD, the membrane is clean with no grooves (a–c). Dissecting the membrane after use in HD to observe the impact of uremic blood on its walls showed the morphology had significantly changed. The presence of networks of fibrous structures, which are consistent with fibrin from blood clots, can be observed. These features are conspicuous on the highest magnification SEM images in the second and third columns (d–i). These fibrin-type clot features could be attributed to insoluble fibrous meshes formed from fibrinogen and are capable of impeding blood flow. Similar images were collected for the PAES hybrid membrane. The pristine PAES membrane has a smooth microtube fiber surface (Fig. 3a1–c1) before HD, but pore blockage with blood/membrane aggregates is evident after HD (Fig. 3d1–i1). For both CTA and PAES, the fouled membranes featured in the middle column show evidence of clotted blood or blood aggregates within the membrane surfaces (d, d1) but more intense pore-blocking features could be observed within the structure of the fiber due to changing membrane chemistry (e, e1 and f, f1). The fouling events on the magnified images (third column, g, g1 to i, i1) are more intense due to aggregation and pore-blocking activities. The cross-section of the microtubes shows they are completely fouled with clots that cover the microporous structures. The CTA was less fouled upon blood contact than the PAES membrane, which is attributed to differences in surface chemistry.

Figure S7 shows TEM cross-section micrographs of pristine and blood-contacted CTA and PAES:PVP HD membranes at low (a and b) and high (c and d) magnifications. These images clearly show porous fiber structures with wider and more extended microtube support structures that contain void channels (exterior surface) and a thin microporous layer (interior surface) [see (a)]. In both cases, the surface illustrations after HD show CTA and PAES–PVP membranes with surface clots and aggregates attached to the respective membrane surfaces (marked in yellow on the images). A lighter and more transparent layer of albumin is also covering the clot. At higher magnifications, the effects of blood-membrane interfacial interactions are evident as well as the attachment of blood-based biomolecules. The TEM cross-section micrograph of the CTA membrane after HD shows less fibrin formation than the PAES membrane (Fig. S7c,d). As identified by the marked spots for PAES in Fig. S7b, extracellular vesicles appear on the pore walls; this appears to be more prevalent in Fig. S7c,d. As these were not observed on the micrograph of the pristine fiber, the existence of vesicles is a direct consequence of blood-fiber interactions and the intra-attachment of biomolecules on the porous membrane walls. Results from these TEM analyses are in good agreement with those for SEM analyses discussed above.

### Molecular docking between polymer ligands and human serum proteins

Blood-material interactions evoke a complex series of interrelated events, including protein adsorption at solid surfaces, platelet and leukocyte activation/adhesion, and regulation of complement activation and blood coagulation. Adsorption of proteins is an instantaneous event that takes place when blood encounters a foreign surface. This layer generally contains proteins, initiating coagulation and thrombotic responses that lead to a change in membrane function. Understanding protein adsorption processes on polymer surfaces requires recognizing the basic structure of proteins to determine the interactions and the binding between a protein and membrane surface. The conformation of adsorbed proteins structure on the membrane surface may play a major role in determining the biocompatibility of a membrane. Disrupted cells can also recruit innate inflammatory cells in the absence of pathogens. Hemolysis induces cell adhesion and increased inflammation. When it comes to disrupted cells, adenosine 5′-diphosphate (ADP) is a key platelet agonist present in erythrocytes and in platelet dense granules. ADP plays a central role in regulating platelet function by the activation of the G protein-coupled receptors P2Y1 and P2Y12. Activation via this receptor leads to the entry of calcium from the extracellular compartment, shape change, and a transient platelet aggregation. Heat shock proteins (Hsp70) are a family of molecular chaperones that promote protein folding. Therefore, Hsp 70 has a dual function as a chaperone and cytokine. Recent studies emphasize that 70 kDa heat shock proteins have higher binding capacities to artificial surfaces^46^.

**Figure 4:**
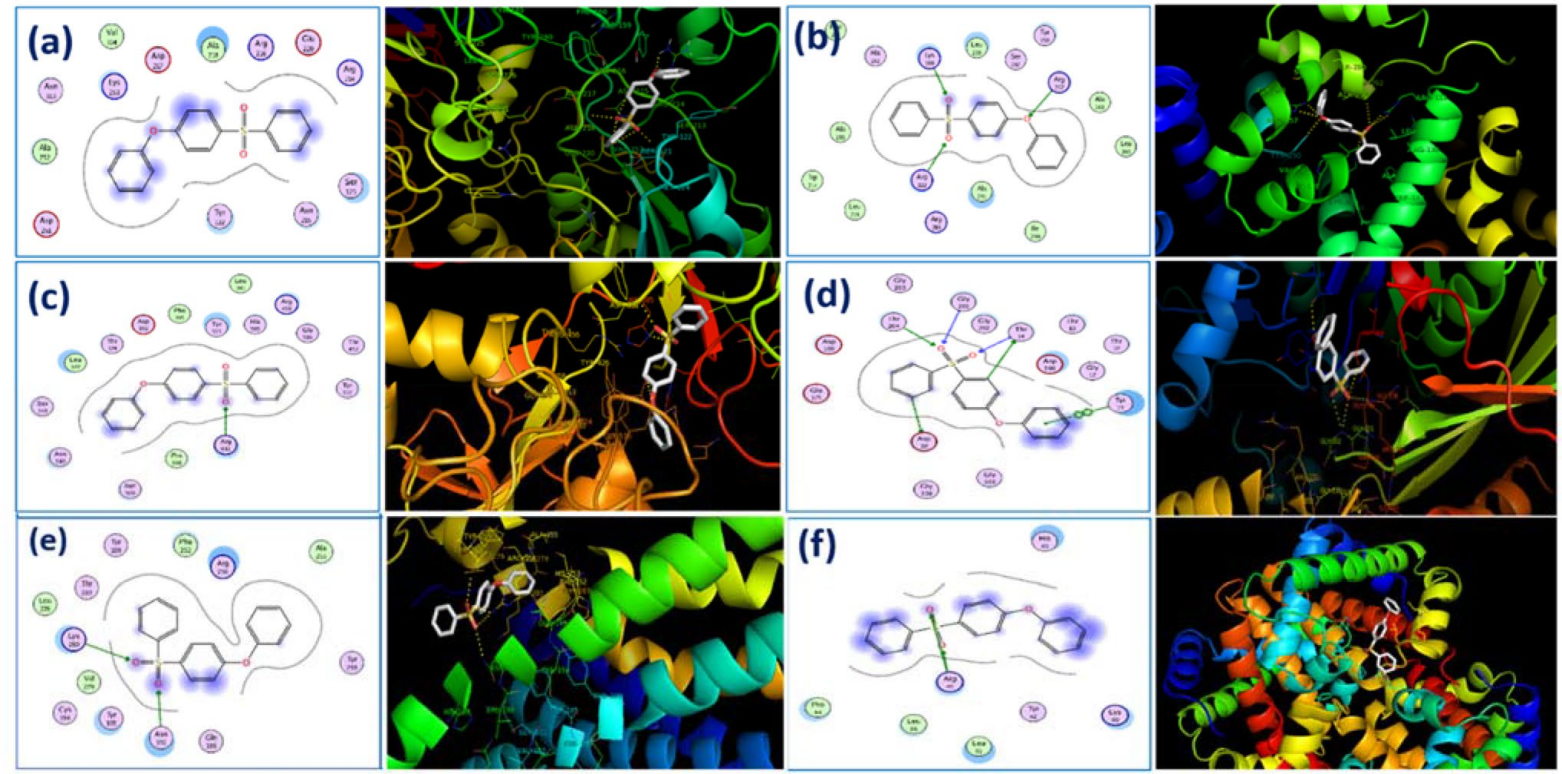
2D (LHS) and 3D (RHS) Molecular docking illustrations showing PAES ligand-proteins interactions for fibrinogen (**a**), albumin (**b**), transferrin (**c**), HSP (**d**), P2Y12 (**e**), and hemoglobin (**f**).

**Figure 5:**
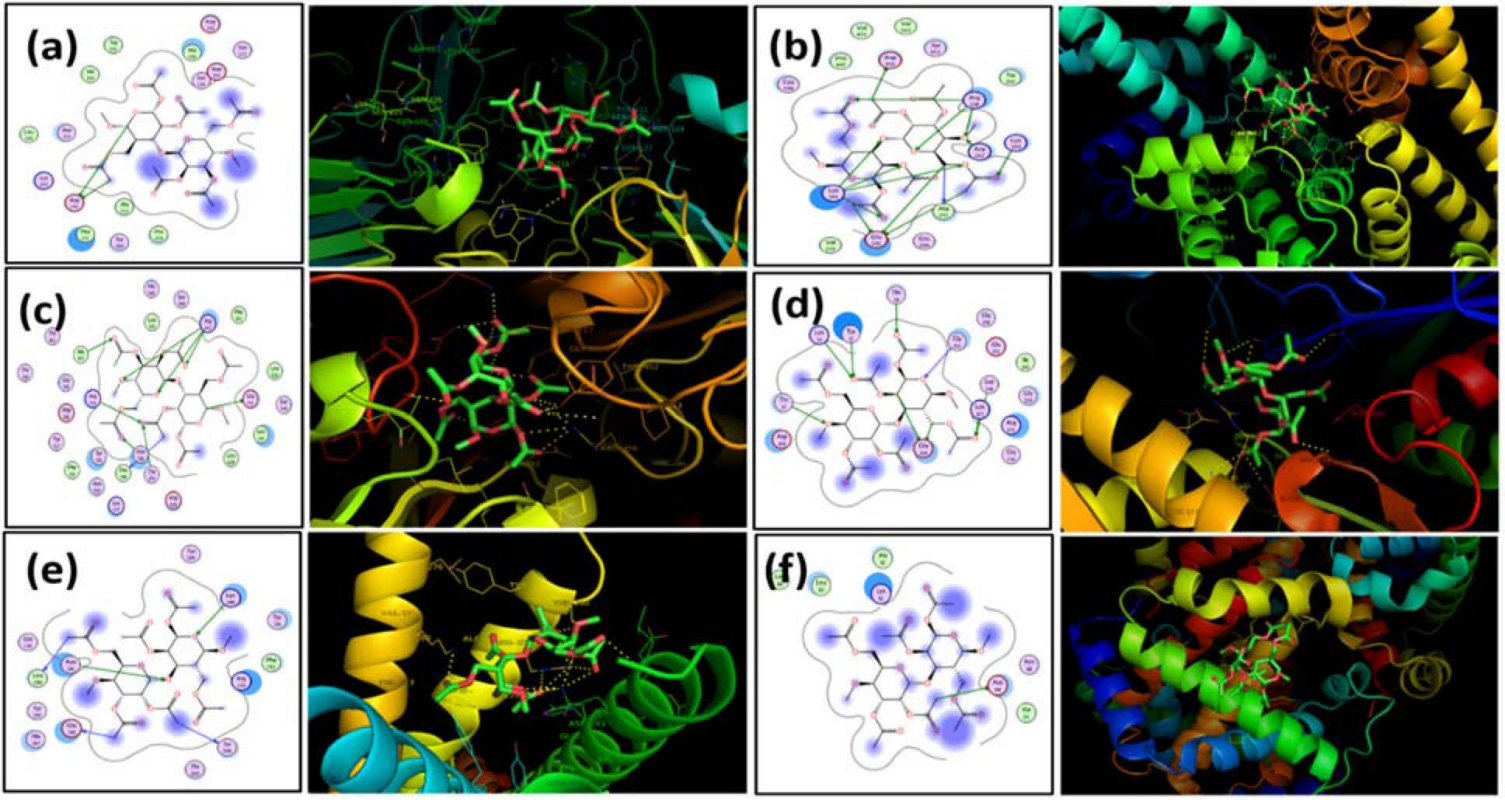
2D (LHS) and 3D (RHS) Molecular docking images showing CTA ligand–protein interactions with fibrinogen (**a**), albumin (**b**), transferrin (**c**), HSP (**d**), P2Y12 (**e**) and hemoglobin (**f**).

Therefore, docking studies considering PAES and CTA ligands and major human serum proteins such as albumin (HSA), FB, transferrin, HSP, P2Y12, and hemoglobin were performed to determine the most favorable binding site with the membranes. On the basis of the top ranked poses, we determined the amino acids to which the monomers preferentially bind as well as the types of interactions taking place. The functional groups on amino acids and monomers dictate the type of noncovalent interaction occurring, be it hydrogen, electrostatic, or hydrophobic. Furthermore, we were able to hypothesize that the CTA monomer ligand is the only ligand that did not have a significant effect on each of the proteins and receptors. Docking studies of the PAES model structure with the active sites of these human serum proteins, based on the X-ray crystallographic structure, exhibited two important interactions: (1) polar contact from the SO_2_ substituent inserts into the hydrophilic pocket of proteins and receptor active sites and (2) hydrophobic interaction from protein residues interacts with phenyl groups in the PAES model. Table S5 summarizes the affinity of each membrane to bind with human serum proteins. Figures 4 and 5 respectively represent 2D and 3D profiles of PAES and CTA ligand–protein interactions, while their corresponding 3D electrostatic profiles are presented in Fig. S8.

Affinity energy ranked results are summarized in Table S5, which shows that PAES binds to active sites of FB, HSA, transferrin, HSP, P2Y12, and hemoglobin with affinities of − 6, − 9.5, − 7.9, − 8.1, − 9.4, and − 5.9 kcal mol^−1^, respectively; CTA binds with lower affinities of − 5.3, − 4.3, − 5.4, − 6.2, − 5.7, and − 1.7 kcal mol^–1^. HSA is a highly helical protein with 585 residues in its primary sequence and 31 α-helices present in its secondary structure. PAES more favorably fit into the hydrophobic cavity. Phe211, His242, Ala215, Trp214, Leu219, Arg218, Ala291, Ile290, Leu260, Ala261, Tyr150, Ser287 and Leu238 were involved in hydrophobic interaction, as presented in Fig. 4. Furthermore, PAES has a hydrophilic interaction with Lys199, Arg222, and Arg257. Thus, the PAES model ligand binds to polar sites of albumin with a high affinity binding interaction. The docking studies reveal the sulfone functional groups of the membranes play an important role in the adsorption of proteins and receptors. These data help to explain the limits to membrane biocompatibility and the intensity change in the spectral analyses.

Figure 5 shows the 2D and 3D (dimensional) structures of CTA with the six proteins of interest located at their most favorable binding sites. Of interest is the fact that CTA preferentially binds at albumin active sites, near residues Asp451, Arg218, Arg222, Lys199, Lys195, and Glu292 (polar contact). Meanwhile, the CTA model ligand’s favorable binding sites with fibrinogen and transferrin proteins were near residue centers Asp232 and Arg632, Glu351, Ala453, Arg456, Asn510, and Thr374, respectively. This indicates the most favorable binding sites are based on polar interactions between oxygen groups from the CTA model and polar functional groups from the amino acids. If this is the case, we would expect CTA to have similar binding contact with hemoglobin (Asp64), HSP (Tyr15), and P_2_Y_12_ (Lys280, Asn191). For both the PAES and CTA model ligands, significant amounts of hydrogen bonding as well as electrostatic and hydrophobic interactions are occurring between the target and ligand.

### Fibrinogen adsorption in CTA vs. PAES–PVP

Fibrinogen plays an important role in many biochemical cascades activated by its adsorption on membrane surfaces, including contributions related hemocompatibility in cases involving thrombosis and embolism^47^. Our theoretical investigation using molecular docking allowed us to observe its affinity for both PAES–PVP and CTA membranes. We compared the adsorption of fibrinogen on the clinical modules of both membranes under the same operating conditions; the inflammatory biomarkers data from patients undergoing HD treatments were used to better understand the consequences of fibrinogen adsorption.

Changes in the normalized concentration of fibrinogen (measured in mg mL^−1^) adsorbed with time during simulated dialysis with each membrane are presented in Fig. S9. The normalized values (Ci/Co) are the ratio of the concentration measured at a specific time (Ci) to the protein concentration (Co) in simulated blood. For the PAES–PVP membrane, a slow increase in the amount of protein adsorbed was observed until it peaked, indicating extreme molecular adsorption. A similar increasing trend was also observed for the CTA membrane but at a faster rate over the first few minutes, subsequently reaching an equilibrium state. Once equilibrium was achieved, the normalized concentration of fibrinogen on the CTA and PAES-PVP membranes was 0.45 and 0.62, respectively. The time to reach equilibrium significantly differed between the two membranes, at 3 min for CTA and 25 min for PAES–PVP. The substantial difference in fibrinogen adsorption behaviour between the two membranes helps to explain the difference in blood activation experienced when these membranes are used in clinical practice.

During the simulated HD session, the transmembrane pressure (TMP) of the PAES–PVP membrane was negative (average over first 10 min was − 0.23 psi), which means that backfiltration occurred. As previously mentioned, PAES–PVP has larger pores that CTA, which increases the risk of backfiltration, which was observed experimentally. In addition to larger pore size, the PAES–PVP membrane is also thicker than CTA. As such, FB molecules likely absorb both within the pores and at the membrane surface. This explains why the adsorption of FB in PAES–PVP was slower compared to CTA, but other factors such as surface roughness, zeta potential, and chemistry contributed to a larger amount of protein adsorbed on the PAES–PVP membrane at saturation.

For the CTA membrane, the TMP was positive during the simulated HD session (average 1.08 psi over the session), with a rapid increase of pressure on the inlet of protein solution observed. This resulted in considerable water loss from the patient side to the dialysate side. Furthermore, higher inlet pressure is expected to result in faster membrane fouling due to the formation of a denser cake layer. This behavior can be associated with the membrane morphology as previously discussed. Specifically, the CTA membrane is considerably thinner than the PAES–PVP membrane and has smaller pores. Additionally, the surface area of the CTA membrane is more than six times higher than the PAES–PVP membrane. As such, the protein layer mainly forms on top of the CTA membrane surface, leading to mostly reversible fouling (proteins keep attaching and detaching from the surface through self-exchange mechanisms). As such, protein molecules can rapidly adhere to the largely available membrane surface leading to an increase in pressure and rapid increase in the amount of FB adsorbed (Fig. S9). The rapid formation of a FB layer also possibly reduced further adsorption, leading to a lower amount adsorbed at saturation. Furthermore, the formation of the FB layer on the CTA surface in the first 3 min may explain how CTA protects patients’ blood from further blood activation and the release of inflammatory biomarkers compared to the PAES membrane. For example, the PAES membrane resulted in continuous adsorption in the first half hour until it achieved equilibrium (Fig. S9), which caused more blood inflammation as noted in the clinical data. A previous study shows that membranes that adhere more FB tend to promote more platelet adhesion^47^. These results indicate the PAES–PVP membrane adsorbs more FB molecules than the CTA membrane at saturation and may explain the many side effects experienced by patients using this membrane, such as back-pressure, which increases the mortality rate.

### Inflammatory biomarkers in HD patients using CTA and PAES

This portion of the research study was performed with the aim of analyzing whether a single dialysis procedure induces complement and inflammatory factors in HD patients, specifically comparing our two dialysis membranes with different biocompatibility features. Our major finding is that a single dialysis session, even with a more biocompatible membrane such as CTA, increases the levels of complement and inflammation factors, but to a milder extent than dialysis with a PAES membrane.

**Figure 6:**
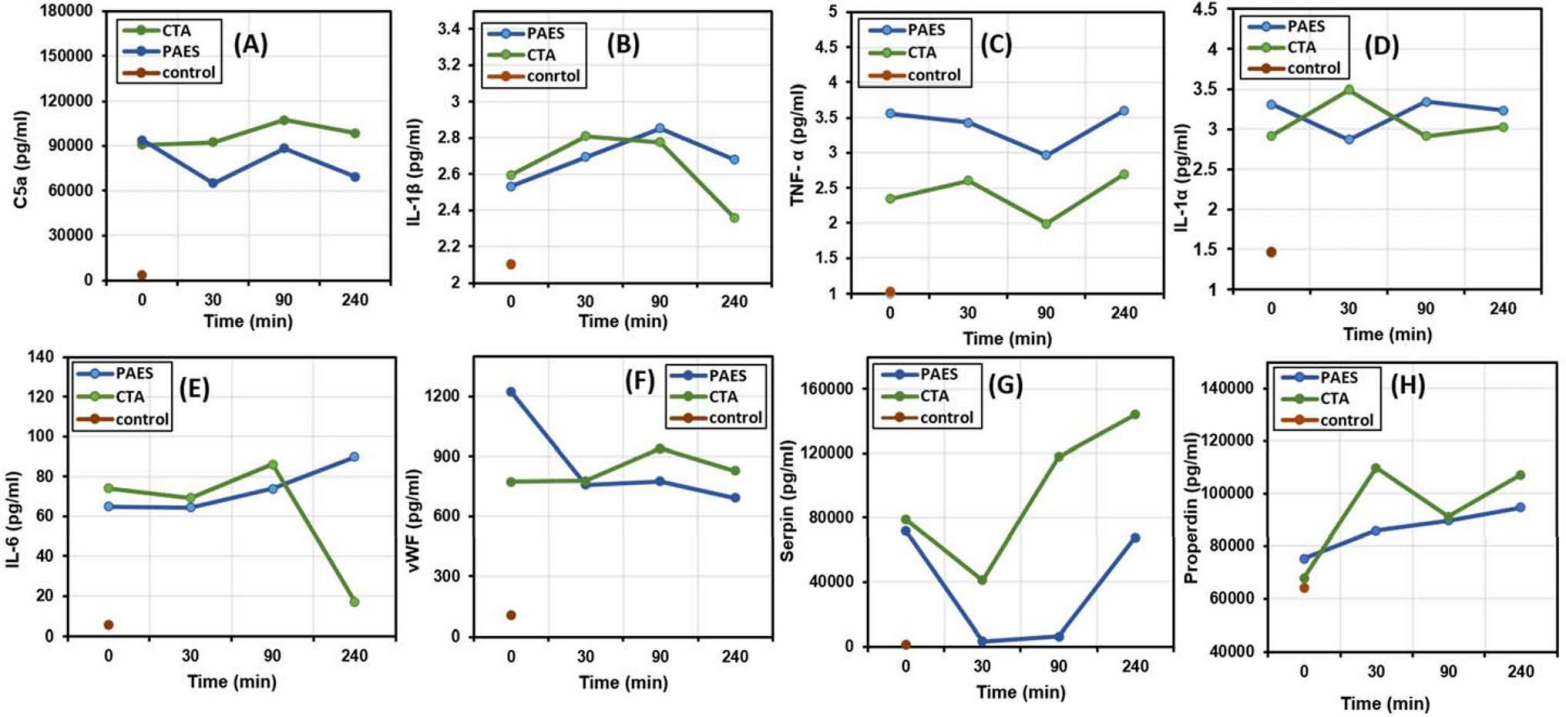
Average Concentrations of cytokines and inflammation factors during HD of healthy controls and HD patients treated with PAES or CTA HD membranes (n = 8). Levels of (**A**) C5a, (**B**) IL-1β, (**C**) TNF-α, (**D**) IL-1α, (**E**) IL-6, (**F**) vWF, (**G**) serpin, and (**H**) properdin throughout a single dialysis procedure. (The concentrations of cytokines and inflammation factors of HD individual patients (n = 8) are presented in Fig. S10).

Clinical data add new complexity to our knowledge and clarify how membrane morphology and chemistry used in the dialyzer can have a significant effect on the levels of the toxins released. Figure 6A–H shows clinical and biochemical characteristics of HD patients and healthy controls. Figure 6A–H shows the average concentration of each cytokine of the HD patient group used CTA or PAES dialyzer vs HD treatment time. Furthermore, each cytokine concentration vs time for each individual patient were provided in the Supplementary information (Fig. S10). Fluctuations in the levels of inflammatory biomarkers in the HD patients are due to their sudden removal at the beginning of dialysis followed by concurrent blood activation and dialysis removal efficiency. To assess complement activation, we determined C5a levels over the course of one HD session. The C5a level before the HD session was not statistically different between patients treated with the CTA membrane (90,637.61 ± 6,855.15) vs. PAES membrane (93,992.63 ± 68,221.845). In eight HD patients, C5a had a higher level of than the two healthy controls before the HD session; however, clear differences between the two HD groups (treated with CTA vs. PAES membranes) were evident both during and after the HD session (Fig. 6A). During the initial 30 min, the C5a concentration decreased by 1.45-fold in the PAES group (P < 0.1) but by only 0.98-fold in the CTA group (P < 0.06). At the end of the HD session, the C5a level was also statistically different between the two groups (CTA group: 98,516.65 ± 16,067.39 pg mL^−1^, PAES group: 69,285.83 ± 38,876.37 pg mL^−1^). Therefore, C5a mean values were significantly higher in HD patients with CTA membrane than in PAES group. We next set out to assess the contribution of the HD-induced complement activation.

Properdin level was also measured in the two groups of patients (Fig. 6H). In those who used the CTA membrane, properdin concentration did not differ significantly between HD patients and healthy controls. At 30 min intradialysis, properdin levels increased significantly in the CTA group (P < 0.09), but it had an insignificant increase in the PAES group (P < 0.008). Conversely, by the end of the dialysis session, properdin levels had significantly increased in the PAES and CTA groups, by 25.90 and 58.06%, respectively (Fig. S11). Overall, properdin levels were comparable between the two groups at the start and end of the HD session despite the opposing patterns observed therein.

Cytokines, inflammation factors, and the vWF were evaluated as inflammatory and pro-thrombotic factors in the HD patients. In the PAES group, vWF levels decreased significantly during the session (P < 0.02). Furthermore, compared to the PAES group, the CTA group had steadily higher levels of vWF at 90 and 240 min intradialysis (P < 0.09) (Fig. 6F). The level of vWF was significantly higher in HD patients compared to controls before, during, and after treatment. Cytokines such as tumor necrosis factor-α (TNF-α) may initiate inflammation. TNF-α levels were significantly higher in patients than controls. Levels of TNF-α rose steadily during the HD session in both groups (Fig. 6C) then dropped 90 min after the start of the HD session. Furthermore, for patients in the PAES group, higher TNF-α levels were observed (P < 0.08) compared to CTA group (P < 0.1). Interestingly, IL-1α levels were steady in both groups at the start of the HD session and the end of dialysis session, although not significant compared to each other (both PAES and CTA had P < 0.06 for IL-1α) (Fig. 6D). IL-1α levels remained higher in HD patients than controls. IL-6 values were significantly higher in patients exhibiting elevated cytokines than healthy controls. Moreover, IL-6 showed an increasing trend during intradialysis to the 90 min mark. At 240 min, an important decrease (by 76.82%) in the IL-6 occurred in the CTA group (P < 0.1), indicating a shift toward a less inflammatory profile (Fig. 6E). However, IL-6 levels remained elevated in HD patients treated with the PAES membrane (increased by 38.67%), contributing a significant difference between the PAES and CTA groups at this time point (P < 0.06). At the start of dialysis session, the CTA and PAES groups had comparable levels of IL-1β (Fig. 6B); however, by the end of the session IL-1β had statistically decreased by 9.20% in the CTA group (P < 0.02) and increased by 5.89% in the PAES group (P < 0.01).

The results obtained show that a dialysis session with a either CTA or PAES membrane can cause increased levels of serpin and properdin. However, the increase was more marked when using CTA vs. PAES due to the high porosity and large pore size of PAES compared to the CTA dialyzer, which led to efficient clearance of middle molecules. Serpin concentrations decreased significantly during the first 30 min of HD (Fig. 6G); for the HD patients, the PAES group (P < 0.4) showed a significantly greater decrease in serpin than the CTA group (P < 0.4). Thereafter, the level of serpin gradually increased to predialysis levels within 90 min of the onset of dialysis due to blood activations. Serpin levels were higher after a dialysis session for the CTA group but remained at predialysis levels for the PAES group. These results are not attributed to the bioincomptability of CTA, but rather its small pore size that results in inefficient toxin clearance. Serpin levels in the controls were lower than in HD patients.

Figure S11 shows the overall percent change in the level of each biomarker under investigation. These changes compare the level of each inflammatory biomarker pre- and post-dialysis (240 min). Some of cytokines coming in contact with the CTA membrane increased after an HD session, with increases of 14.92, 3.82, 8.69, 7.06, 82.61, and 58.06% for TNF-α, IL-1α, C5a, vWF, serpin, and properdin, respectively. The levels of IL-1β and IL-6 significantly declined (by 9.20 and 76.82%) for the CTA membrane. Alternatively, C5a, IL-1α, vWF, and serpin levels decline in HD patients treated with the PAES membrane (by 26.29, 2.33, 43.34, and 6.10%, respectively) (Fig. S11). These results indicate the increase in cytokines and inflammatory factors after a dialysis session is due to circulating blood contact with membrane biomaterials and the morphology of the membrane, thus triggering the complement and coagulation cascades. The goal of our study was the collection of data that could contribute to a better understanding of the processes occurring on HD membranes when coming in contact with blood. The adsorption of proteins onto the HD membrane is one of the initial events occurring upon blood contact. The adsorption process has unclear effects on the adhesion of platelets or activation of the coagulation and complement system. Unfortunately, inflammatory biomarkers were not suppressed to the levels noted in controls for any of the patient test cases. In all of these instances, levels of inflammatory biomarkers either remained at almost the same level or increased to levels higher than before dialysis treatment due to severe biological activations. The exceptions are IL-1β and IL-6 in patients treated with the CTA membrane and C5a, IL-1α, and vWF in patients treated with the PAES membrane. These contradictory results indicate each dialysis membrane had its own overall advantages in terms of biocompatibility impact and clearance.

Note that the PAES membrane’s high negative surface charge of − 68 mV is double that of CTA. This higher charge makes it a more hydrophilic membrane that in turn causes the absorption of water from the blood flow. Therefore, the dehydration of RBCs directly influences the concentration of RBCs in plasma, leading to a higher probability of RBC rupture and hemolysis. This dynamic adversely affects the immune system, causing the release of more cytokines and leukocytes, and resulting in patients treated with the PAES membrane exhibiting higher levels of IL-1β, IL-6, and TNF-α.

On the one hand, the use of CTA membranes with smaller pore sizes can effectively remove small toxins. This may explain the higher levels of serpin and properdin in patients who using the CTA dialyzer vs. PAES dialyzer. On the other hand, the use of PAES membranes with larger pores compared to CTA allows the system to operate at high flux and promotes the clearance of middle molecules and protein-bound toxins. However, the use of PAES might lead to the depletion of physiological proteins and increase the potential for backfiltration, which in turn can reintroduce toxins from the dialysate back into the blood stream^45^.

### In vitro incubation of membrane materials in uremic blood

**Figure 7:**
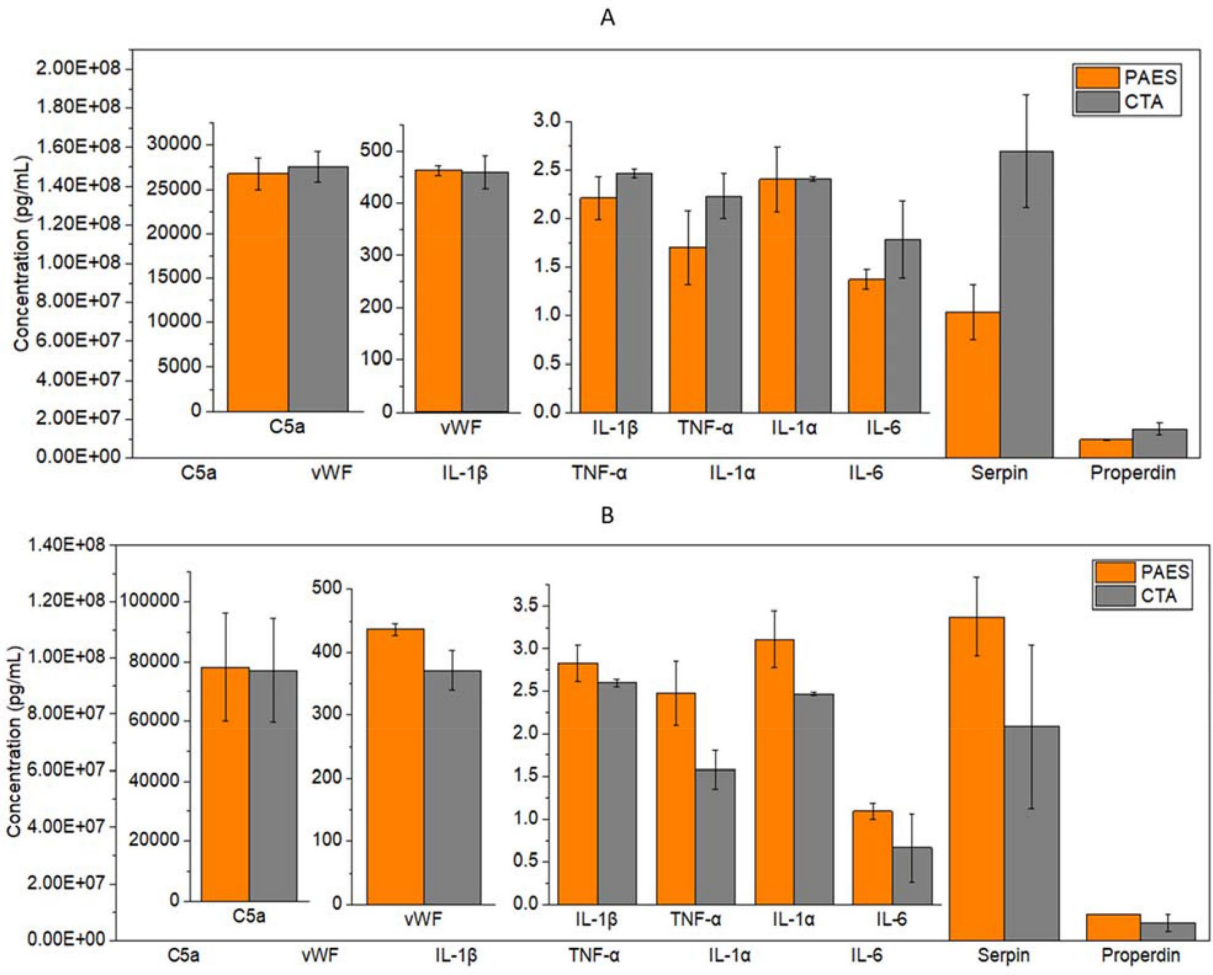
Levels of cytokines and inflammation factors during in vitro incubation of CTA and PAES membrane in uremic serum from a patient normally treated with (**A**) PAES dialyzers; and (**B**) CTA dialyzers. In each case, the samples were collected prior to the patients’ regular treatment (n = 3) and then incubated in vials with each membrane type (3 samples each) for 30 min. Data points and error bars represent the mean ± standard error of the mean.

Our research group was interested in differentiating between the influence of hydrodynamics during dialysis treatment and the hemo-compatibility of the membrane materials used. Three samples of PAES and CTA membranes were incubated serum samples from patients normally treated with either CTA and PAES dialyzers (n = 3), to eliminate the hydrodynamic influence, as well as the side effects of the shear stress and the ruptured blood cell due to higher roughness. In addition to investigating how the membrane material of specific dialyzer can trigger more blood activations of patients normally treated with the opposite membrane. The complement and coagulation activation that can be attributed to the HD membranes was investigated by incubating pieces of CTA and PAES membranes in patient serum, normally treated with either CTA and PAES dialyzers, for 30 min, then measuring the cytokines and inflammation factors released. Figures 7A,B presents the results of the incubation of PAES and CTA membranes in serum samples from patients normally treated with either PAES and CTA dialyzers, respectively. As presented in Fig. 7A, incubating PAES membrane in the serum of patients normally treated with PAES (n = 3), had mild results of inflammatory biomarkers compared to actual HD results, which are comparable to the biocompatible CTA (n = 3). The mild results are attributed to the elimination of the hydrodynamic influence, the side effect of shear stress, and the rupture of blood cells due to higher roughness of the PAES membrane compared to the CTA membrane. This process activated one time the levels of C5a, IL-1β, IL-1α, IL-6, and properdin than those exposed to PAES, and approximately two times the levels of antithrombin III bound to the CTA membranes. These mild results are attributed to the elimination of the hydrodynamic influence, the side effect of shear stress, and the rupture of blood cells due to higher roughness of the PAES membrane compared to the CTA membrane.

However, as shown in Fig. 7B, TNF-α and vWF concentrations were greater when serum samples from patients normally treated with CTA incubated with the PAES membrane. Based on the patient case study measurements, PAES membranes are classified as strong coagulation activators compared to CTA membranes. We likewise concluded that the amount of fibrinogen adsorbed in the presence of PAES was higher than the quantity adsorbed in the presence of CTA. Serums of patients using CTA dialyzers and exposed to PAES membranes induce more cytokines and inflammation factors, confirming the better biocompatibility of CTA. This decrease in activation with CTA suggests PAES is a potent complement and coagulation activator. Thus, complement and coagulation activating potential is a critical criterion that can be used to determine the biocompatibility of membranes.

## Conclusions

The goal of the present study was to conduct a series of in-depth investigations on selected clinical HD membranes available in Canadian hospitals, with a view to exploring key reasons behind their susceptibility to blood activation and unstable cytokine concentrations. The following key findings are drawn from the experimental results of all tests conducted.

- CTA membranes have a smoother surface, smaller fiber diameter, and thinner fiber wall than PAES:PVP membranes. Furthermore, CTA fibers have a small pore size distribution that results in poor clearance of a broad spectrum of uremic toxins. CTA membranes have a unique lined structure at their surface that promotes better flow distribution.
- PAES membranes have a rougher surface, larger pore size a broader pore size distribution, and a thicker fiber wall than CTA membranes. The rougher surface is associated with increased red blood cell rupture at the membrane surface, which promotes protein adsorption and biochemical cascade reactions. The broad pore size distribution promotes better clearance of a broad spectrum of uremic toxins.
- PAES is notably more hydrophilic than CTA. The superior hydrophilicity is associated with red blood cell dehydration and rupture, which can lead to severe patient outcomes. The major difference between the chemical composition of the membranes is the trace amounts of S and N found in PAES-PVP. These elements are associated with decreased biocompatibility. Hence, PAES is less hemocompitible, but the larger pore size allows for better clearance. Upon contact with uremic blood, PAES demonstrates a higher propensity for fouling compared to CTA.
- When compared to CTA, PAES presents higher (more negative) affinity energy to FB, HSA, transferrin, HSP, P2Y12, and hemoglobin. Docking studies indicate the sulfone functional groups of the membranes play an important role in the adsorption of proteins and receptors. Major polar and hydrophobic interactions from protein residues occurred with phenyl groups in the PAES model.
- PAES-PVP membranes result in slower but greater adsorption of FB compared to CTA membranes. In in vitro experiments, PAES membranes experienced backfiltration, which is undesirable in clinical practice. PAES-PVP is more likely to experience reversible and irreversible fouling due to the large pore size distribution. Other morphological and chemical properties of PAES-PVP membrane contributed to higher amount of FB adsorbed.
- A single dialysis session, even with a biocompatible membrane such as CTA, increases the levels of complement and inflammation factors, but to a milder extent than PAES dialysis.
- Serum incubation in PAES had mild results because we eliminated the hydrodynamic influence, as well as the side effects of the shear stress and the ruptured blood cell due to higher roughness of PAES membrane, compared to the CTA.
- Serums of patients using CTA dialyzers exposed to PAES membranes have been shown to induce one time the amount of all cytokines and inflammation factors, thus confirming higher biohemocomtability of CTA.
- The decrease in activation when using CTA suggests that PAES is a potent complement and coagulation activator.
- Our research findings significantly contribute towards the understanding of why patients experience side effects when undergoing HD treatment. The correlations outlined here can help clinical doctors to investigate how patients’ serum can allow for more blood activations in specific modules, before prescribing the dialysis modules.

## Supplementary information

Supplementary Information.

## Data Availability

The raw/processed data required to reproduce these findings cannot be shared at this time, as the data are critical to ongoing research.
