# Supplementary material for: Case studies of clinical hemodialysis membranes: influences of membrane morphology and biocompatibility on uremic blood-membrane interactions and inflammatory biomarkers
Source: Sci Rep. 2020 Sep 9;10:14808. doi: 10.1038/s41598-020-71755-8 (PMC7481195; doi:10.1038/s41598-020-71755-8)
Supplement: Supplementary file 1 — Supplementary Information. [file 41598_2020_71755_MOESM1_ESM.docx]

**Case Studies of Clinical Hemodialysis Membranes: Influences of Membrane Morphology and Biocompatibility on Uremic Blood-Membrane Interactions and Inflammatory Biomarkers**

Heloisa Westphalen^1^, Shaghayegh Saadati^1,2^, Ubong Eduok^1^, Amira Abdelrasoul^1,2*,^ Ahmed Shoker^3,4^, Phillip Choi^5^, Huu Doan^6^, Farhad Ein-Mozaffari^6^

*^1^Department of Chemical and Biological Engineering, University of Saskatchewan, 57 Campus Drive, Saskatoon, Saskatchewan, S7N 5A9, Canada.*

*^2^ Division of Biomedical Engineering, University of Saskatchewan, 57 Campus Drive, Saskatoon, Saskatchewan, S7N 5A9, Canada.*

*^3^ Nephrology Division, College of Medicine, University of Saskatchewan, 107 Wiggins Rd, Saskatoon, SK S7N 5E5*

*^4^ Saskatchewan Transplant Program, St. Paul's Hospital, 1702 20th Street West Saskatoon Saskatchewan S7M 0Z9 Canada*

*^5^Department: Chemical and Materials Engineering, University of Alberta, 243 Donadeo Innovation Centre For Engineering, Edmonton, Alberta, T6G 2H5, Canada*

*^6^Department of Chemical Engineering, Ryerson University, 350 Victoria Street, Toronto, Ontario,*

*M5B 2K3, Canada*

****Corresponding Author: amira.abdelrasoul@usask.ca, Tel: (306) 966 2946, Fax: (306) 966 4777***

**SUPPORTING INFORMATION**


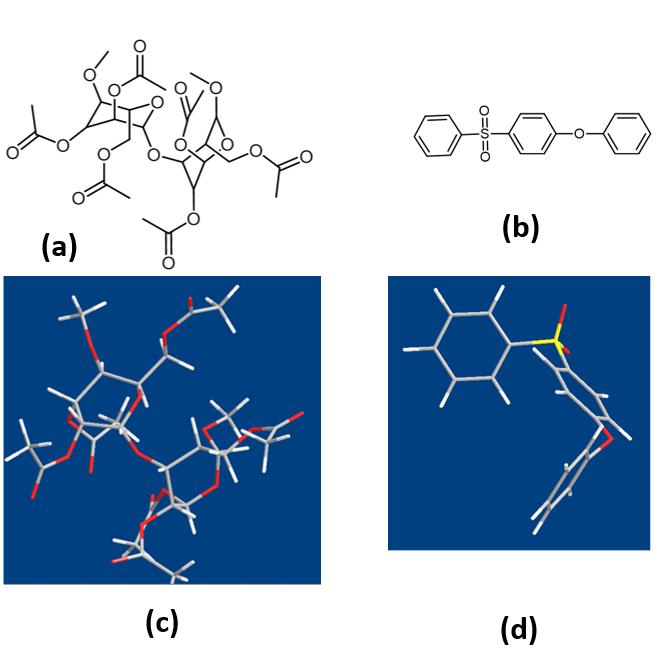


**Figure S1.** Molecular (a.b) and optimized molecular (c,d) structures of CTA (a,c) and PAES (b,d)


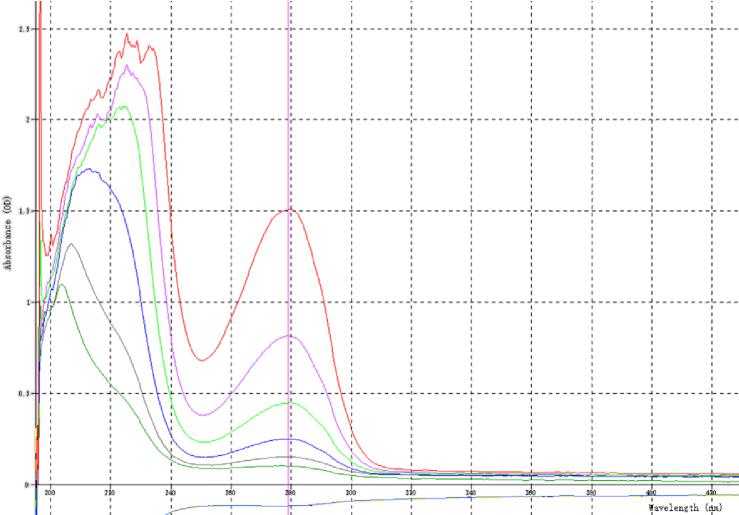


**Figure S2.** UV/Vis spectra of FB in PBS solution


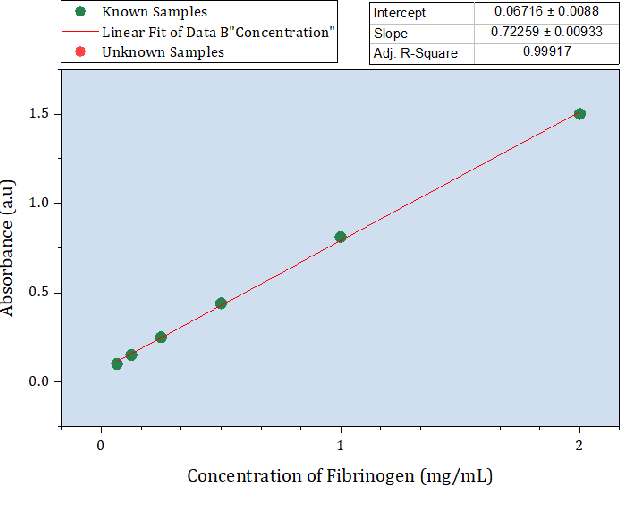


**Figure S3.** Calibration curve for FB concentration using absorbance at 278.906 nm.


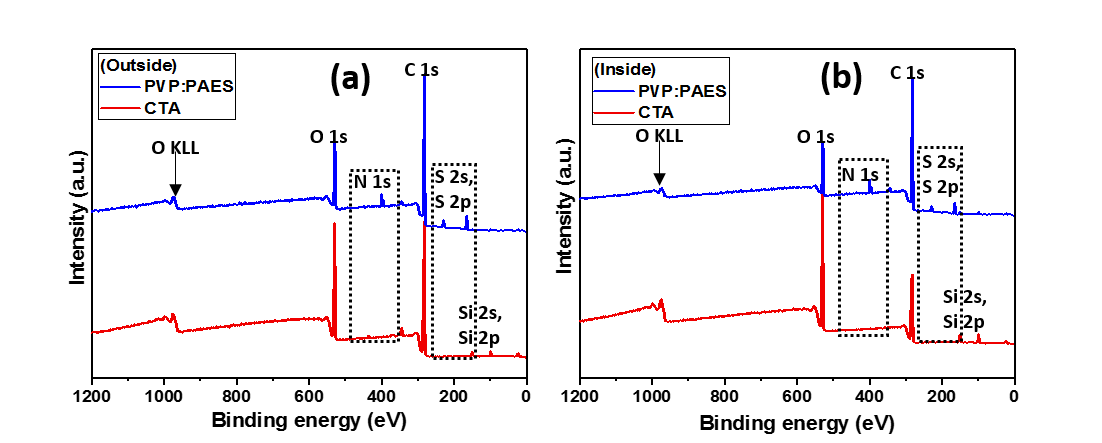


**Figure S4.** XPS wide-scan spectra of the outside (a) and inside (b) of CTA and PAES polymer membrane fibers.


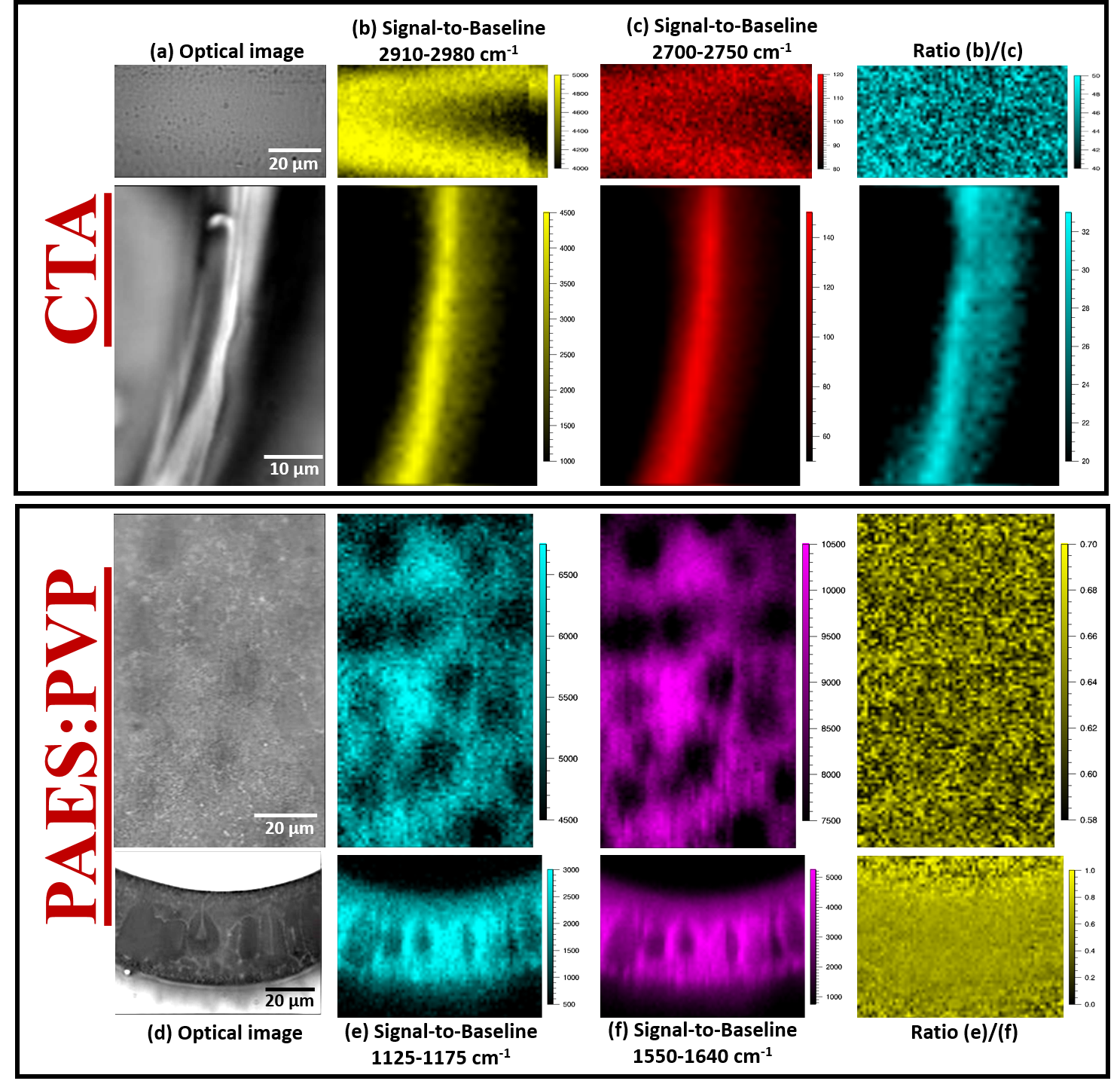


**Figure S5.** Optical images and Raman mapping of the surface (first and third rows) and cross-section (second and fourth rows) of CTA and PAES:PVP hollow membrane fibers.


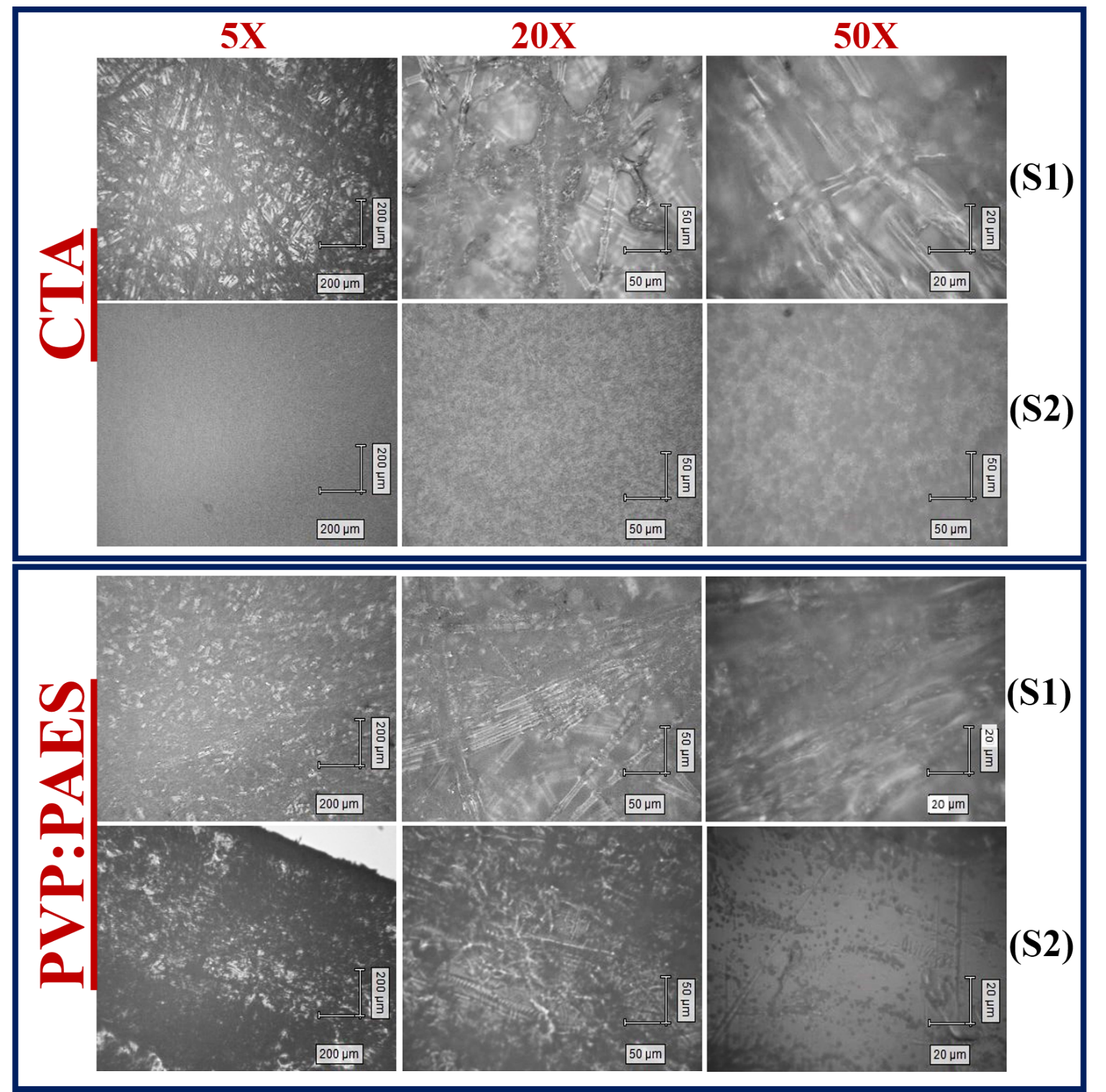


**Figure S6.** Raman mapping micrographs of the inside (S1) and outside (S2) of CTA and PVP:PAES polymer membrane fibers.


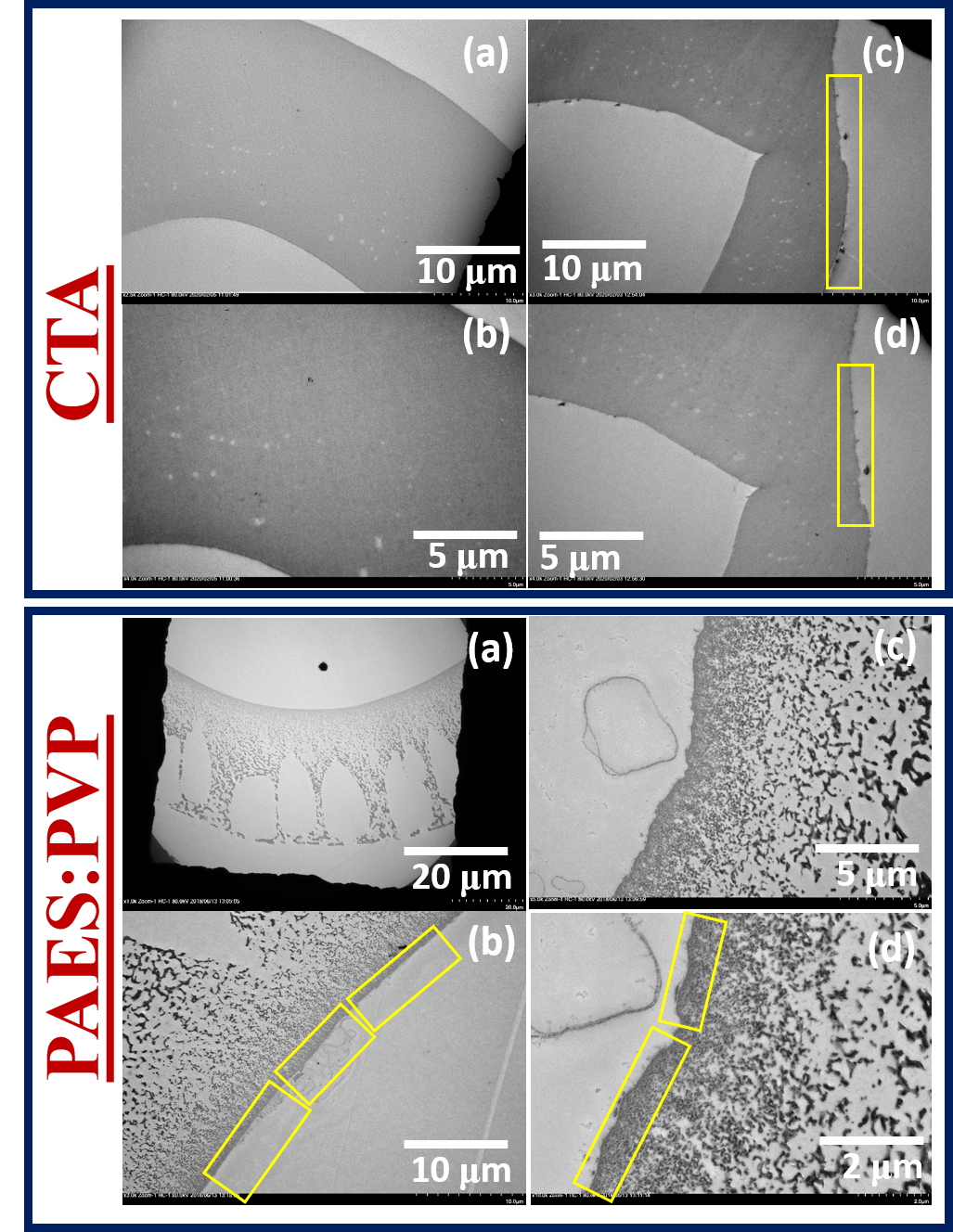


**Figure S7.** TEM cross-section micrographs of pristine and blood-contacted CTA and PAES:PVP hemodialysis membranes at low (a and b) and high (c and d) magnifications.


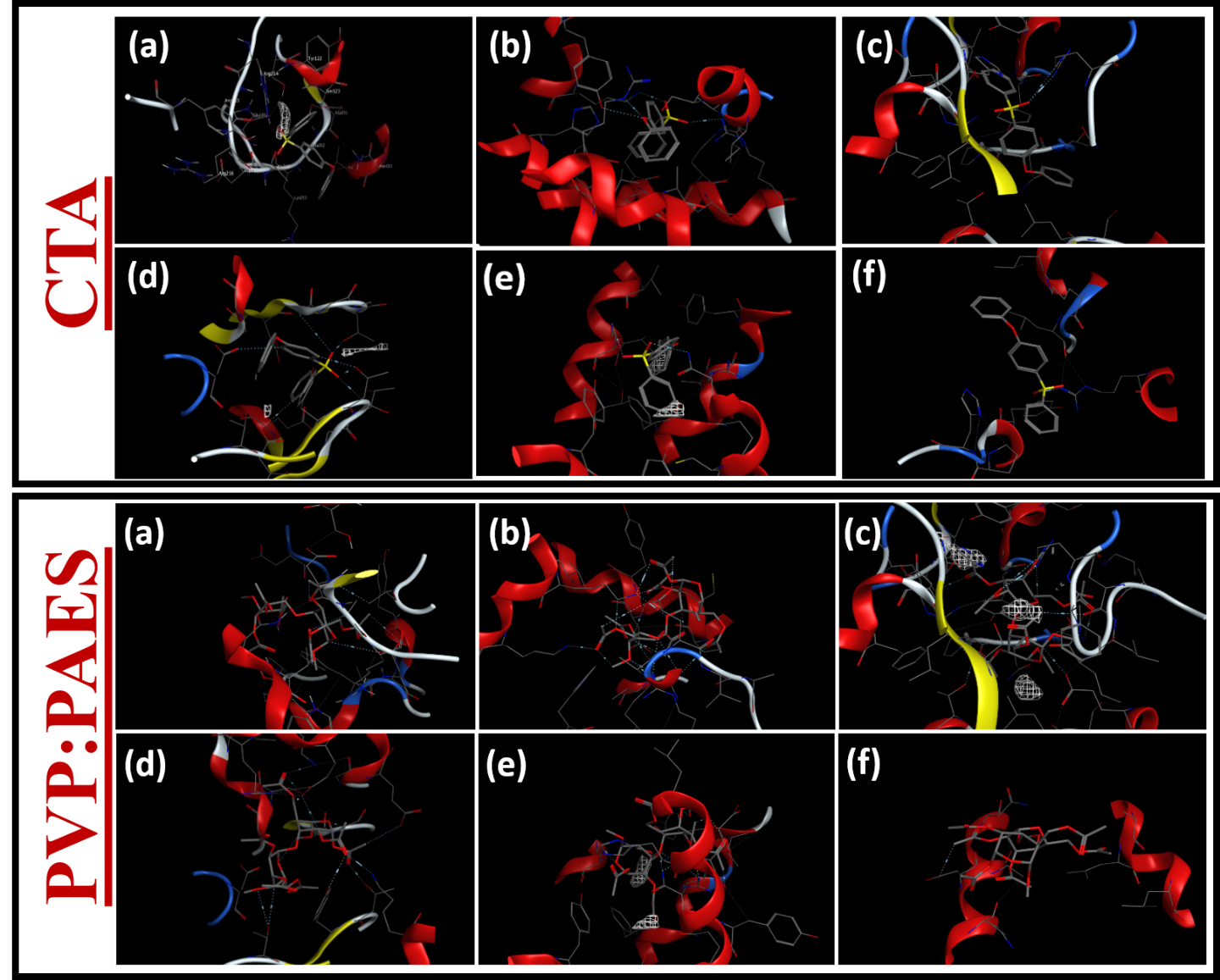


**Figure S8.** Electrostatic profile of CTA and PAES (by Molecular Operating Environment, MOE) and selected proteins: FB (a), albumin (b), transferrin (c), HSP (d), P2Y12 (e), and hemoglobin (f).

**Figure S9.** FB adsorption on CTA/PAES-PVP under conditions simulating typical dialysis (blood flow rate, 300 mL min^-1^; dialysate flow rate, 500 ml min^-1^)

**(a)**

**(b)**

**(c)**

**(d)**

**(e)**

**(f)**

**(g)**

**(H)**

**Figure S10.** Concentrations of cytokines and inflammation factors of HD individual patients (n=8). Levels of (A) C5a, (B) IL-1β, (C) TNF-α, (D) IL-1α, (E) IL-6, (F) vWF, (G) serpin, and (H) properdin throughout a single dialysis procedure.

**Figure S11.** Overall percent change in the concentration of inflammatory biomarkers for the CTA and PAES patient groups (n=8) at the end of HD treatment. Data points and error bars represent the mean ± standard error of the mean.

**Table S1.** Composition of dialysate solution.

| Ions | Concentration (meq L^-1^) |
| --- | --- |
| Na^+^ | 132-145 |
| K^+^ | 0-4 |
| Cl^─^ | 103-110 |
| HCO_3_^─^ | 0-40 |
| Acetate | 2-37 |
| Ca^2+^ | 0-3.5 |
| Mg^2+^ | 0.5-1 |
| Glucose | 0-200 mg dL^-1^ |

**Table S2.** Surface roughness measurements for hollow polymer membrane fibers.

| Parameter | CTA | | PVP:PAES | |
| --- | --- | --- | --- | --- |
|  | Inside  (2 × 2 µm)  (n = 3) | Outside  (2 × 2 µm)  (n = 4) | Inside  (2 × 2 µm)  (n = 3) | Outside  (1.5 × 1.5 µm)  (n = 4) |
| R_a_ (nm) | 5.4 ± 1.9 | 5.4 ± 1.9 | 10.4 ± 4.0 | 10.5 ± 5.0 |
| R_RMS_ (nm) | 7.5 ± 3.0 | 7.5 ± 3.0 | 15.3 ± 5.9 | 15.4 ± 6.8 |

**Table S3.** BET surface area and zeta potential values for CTA and PAES polymer membrane fibers

| Fiber | BET surface area (m^2^ g^-1^) | Zeta potential (mV) |
| --- | --- | --- |
| CTA | 14.47 ± 1.48 | –34 |
| PAES:PVP | 1.99 ± 0.32 | –68 |

**Table S4.** Percent (%) abundance of elements within the membrane fibers

| Fiber | Morphology | Element (%) | | | | | | |
| --- | --- | --- | --- | --- | --- | --- | --- | --- |
|  |  | O 1s | C 1s | Si 2 p | Ca 2p | N 1s | S 2p | B 1s |
| CTA | Inside | 29.30 | 64.37 | 6.33 | — | — | — | — |
|  | Outside | 21.97 | 74.51 | 2.50 | 1.02 | — | — | — |
| PAES:PVP | Inside | 12.29 | 75.60 | 1.60 | — | 2.08 | 3.83 | 4.60 |
|  | Outside | 13.56 | 76.47 | 0.96 | — | 1.97 | 4.39 | 2.65 |

**Table S5.** Affinity values for ligand-proteins interactions determined from molecular docking studies

| Protein | *K* affinity  (Kcal mol^-1^) | |
| --- | --- | --- |
|  | PAES | CTA |
| Fibrinogen | -6.00 | -5.3 |
| Albumin | -9.5 | -4.3 |
| Transferrin | -7.9 | -5.4 |
| HSP | -8.1 | -6.2 |
| P2Y12 | -9.4 | -5.7 |
| Hemoglobin | -5.9 | -1.7 |

HUMAN MAGNETIC LUMINEX ASSAY – SERPIN/ ANTITHROMBIN-III

Materials

- Pipettors (single and multi-channel)
- Pipette tips
- Orbital shaker
- Microcentrifuge
- Microcentrifuge tubes, 1.5 mL
- Handheld Magnetic Plate Washer (Thermo Fisher, Mississauga, ON)
- Custom magnetic Luminex Assay Kit (Cat #: LXSAHM)
- Bioplex 200
- Bioplex Manager Software

Method

1. Take Magnetic Luminex Assay kit out of the fridge and allow to come to room temperature.
2. Take samples out of the freezer and allow to thaw.

*Preparation of Samples*

1. Spin samples down at 16,000*g* for 4 min.
2. Dilute the samples 1:4000 (as per the Certificate of Analysis)
3. 10 uL of 1:200 diluted serum sample in 190 uL Calibrator Diluent RD6-52

*Preparation of Standards*

1. Reconstitute one vial of the provided Human Standard F cocktail with 250 uL of Calibrator Diluent RD6-52 (refer to Certificate of Analysis)
2. Allow the standard to sit with gentle shaking for at least 15 min.
3. Add 100 uL of Standard R solution to a tube containing 900 uL of Calibrator Diluent RD6-52. This is Standard 1.
4. To five 1.5-mL microcentrifuge tubes add 200 uL of Calibrator Diluent RD6-52. Label these tubes as Standards 2 to 6.
5. Mix Standard 1 by pipetting up and down three times.
6. Transfer 100 uL of Standard 1 into the tube for Standard 2 containing 200 uL of Calibrator Diluent RD6-52.
7. Mix Standard 2 thoroughly by pipetting up and down three times.
8. Transfer 100 uL of Standard 2 to the tube for Standard 3 containing 200 uL of Calibrator Diluent RD6-52.
9. Continue this procedure until you get to Standard 6. Do not transfer any material from the Standard 6 tube (the volume of this tube should be 300 uL)
10. Calibrator Diluent RD6-52 will serve as the blank for the assay.

*Preparation of Microparticle Cocktail*

1. Briefly centrifuge the microparticle cocktail vial for 30 s at 1000*g*.
2. Resuspend the particles by gently vortexing the vial. Do not invert the vial!
3. Dilute the microparticle cocktail using Diluent RD2-1 in the mixing bottle provided.
4. 500 uL of microparticle cocktail + 5.00 mL of Diluent RD2-1

4. Be sure to keep the microparticles protected from light during handling.

*Assay Procedure*

1. To the 96 well plate, add 50 uL of standard or sample to the appropriate wells. Samples and standards are assayed in duplicate.

2. Resuspend the diluted microparticle cocktail by gently mixing the bottle. Transfer the cocktail to a pipette basin and cover with tinfoil to protect from light.

3. Using a multichannel pipette, add 50 uL of microparticle cocktail to each well. Be sure to mix contents of the pipette basin regularly by gently rocking back and forth so particles do not settle to the bottom of the basin.

4. Cover the plate with a foil plate sealer.

5. Incubate the plate at room temperature for 2 h at 800 rpm using an orbital shaker.

6. Remove the plate from the orbital shaker and place it on the handheld magnetic plate washer. Ensure the plate is secure before beginning any wash steps.

7. Allow the plate to sit for 1 min so the microparticles can settle to the bottom of the well.

8. After 1 min, remove the liquid quickly by inverting the plate and magnetic plate washer over the sink.

9. Add 100 uL of wash buffer to each well. Allow to sit for 1 min. Cover the plate with tin foil between each wash.

10. After 1 min, remove the liquid from the plate. Repeat the addition of wash buffer (step 9) to the plate. Repeat the wash procedure for a total of three times.

11. Prepare diluted Biotin Antibody cocktail. Gently vortex the vial to mix.

1. 500 uL of concentrated Biotin Antibody + 5.00 mL of Diluent RD2-1
2. Mix gently.

12. Transfer the diluted Biotin Antibody cocktail to a new pipette basin.

13. Using a multichannel pipette, add 50 uL of diluted Biotin Antibody Cocktail to each well.

14. Cover the plate with a foil plate sealer.

15. Incubate the plate at room temperature for 1 h at 800 rpm on an orbital shaker.

16. Repeat steps 6 to 10 (wash steps).

17. Prepare diluted Streptavidin-PE. Gently vortex the vial to mix.

1. 220 uL Streptavidin-PE concentrate + 5.35 mL wash buffer
2. Mix gently.
3. Cover with tin foil to protect from light.

18. Transfer the diluted Streptavidin-PE to a new pipette basin and cover with tin foil when not in use.

19. Using a multichannel pipette, add 50 uL of diluted Streptavidin-PE to each well.

20. Cover the plate with a foil plate sealer.

21. Incubate the plate at room temperature for 30 min at 800 rpm on an orbital shaker.

22. Repeat steps 6 to 10 (wash steps).

23. Add 100 uL of wash buffer to each well. Cover the plate with a foil plate sealer.

24. Incubate the plate at room temperature for 2 min at 800 rpm on an orbital shaker.

25. Read the plate using the Bioplex 200.

*Setting up Bioplex 200 for Assays*

1. Prepare assay protocols in software before beginning the assay.

2. Ensure the instrument has been validated recently. If not, validate the instrument using the MCV plate and Validation kit (Bio-Rad, Mississauga, ON, Cat # 171203001)

3. On the day of the assay:

1. Warm up the instrument during the 2- or 1-h incubation. Instrument warmup takes approximately 30 min.
2. Calibrate the instrument before the assays using the MCV plate and calibration standards (Bio-Rad, Mississauga, ON; Cat #171203060

4. After calibration has been completed, open the appropriate protocol and run it to begin reading the plate. Be sure the following parameters on the software are inputted correctly.

1. 50 beads/region
2. Appropriate bead region for the analyte (refer to Certificate of Analysis; this should be done when setting up the protocol).

5. Export all data to Excel for analysis.

6. After the plate is read, shut down the instrument. Shut down takes approximately 10 min.

HUMAN MAGNETIC LUMINEX ASSAY – PROPERDIN

Materials

- Pipettors (single and multi-channel)
- Pipette tips
- Orbital shaker
- Microcentrifuge
- Microcentrifuge tubes, 1.5 mL
- Handheld Magnetic Plate Washer (Thermo Fisher, Mississauga, ON)
- Custom magnetic Luminex Assay Kit (Cat #: LXSAHM)
- Bioplex 200
- Bioplex Manager Software

Method

1. Take Magnetic Luminex Assay kit out of the fridge and allow to come to room temperature.
2. Take samples out of the freezer and allow to thaw.

*Preparation of Samples*

1. Spin samples down at 16,000*g* for 4 min.
2. Dilute the samples 1:200 (as per the Certificate of Analysis)
3. 1 uL of serum sample in 199 uL Calibrator Diluent RD6-52

*Preparation of Standards*

1. Reconstitute one vial of the provided Human Standard R cocktail with 300 uL of Calibrator Diluent RD6-52 (refer to Certificate of Analysis)
2. Allow the standard to sit with gentle shaking for at least 15 min.
3. Add 100 uL of Standard R solution to a tube containing 900 uL of Calibrator Diluent RD6-52. This is Standard 1.
4. To five 1.5-mL microcentrifuge tubes add 200 uL of Calibrator Diluent RD6-52. Label these tubes as Standards 2 to 6.
5. Mix Standard 1 by pipetting up and down three times.
6. Transfer 100 uL of Standard 1 into the tube for Standard 2 containing 200 uL of Calibrator Diluent RD6-52.
7. Mix Standard 2 thoroughly by pipetting up and down three times.
8. Transfer 100 uL of Standard 2 to the tube for Standard 3 containing 200 uL of Calibrator Diluent RD6-52.
9. Continue this procedure until you get to Standard 6. Do not transfer any material from the Standard 6 tube (the volume of this tube should be 300 uL)
10. Calibrator Diluent RD6-52 will serve as the blank for the assay.

*Preparation of Microparticle Cocktail*

1. Briefly centrifuge the microparticle cocktail vial for 30 s at 1000*g*.
2. Resuspend the particles by gentle vortexing the vial. Do not invert the vial!
3. Dilute the microparticle cocktail using Diluent RD2-1 in the mixing bottle provided.
4. 500 uL of microparticle cocktail + 5.00 mL of Diluent RD2-1

4. Be sure to keep the microparticles protected from light during handling.

*Assay Procedure*

1. To the 96 well plate, add 50 uL of standard or sample to the appropriate wells. Samples and standards are assayed in duplicate.

2. Resuspend the diluted microparticle cocktail by gently mixing the bottle. Transfer the cocktail to a pipette basin and cover with tinfoil to protect from light.

3. Using a multichannel pipette, add 50 uL of microparticle cocktail to each well. Be sure to mix contents of the pipette basin regularly by gently rocking back and forth so particles do not settle to the bottom of the basin.

4. Cover the plate with a foil plate sealer.

5. Incubate the plate at room temperature for 2 h at 800 rpm using an orbital shaker.

6. Remove the plate from the orbital shaker and place it on the handheld magnetic plate washer. Ensure the plate is secure before beginning any wash steps.

7. Allow the plate to sit for 1 minute so the microparticles can settle to the bottom of the well.

8. After 1 min, remove the liquid quickly by inverting the plate and magnetic plate washer over the sink.

9. Add 100 uL of wash buffer to each well. Allow to sit for 1 min. Cover the plate with tin foil between each wash.

10. After 1 min, remove the liquid from the plate. Repeat the addition of wash buffer (step 9) to the plate. Repeat the wash procedure for a total of three times.

11. Prepare diluted Biotin Antibody cocktail. Gently vortex the vial to mix.

1. 500 uL of concentrated Biotin Antibody + 5.00 mL of Diluent RD2-1
2. Mix gently.

12. Transfer the diluted Biotin Antibody cocktail to a new pipette basin.

13. Using a multichannel pipette, add 50 uL of diluted Biotin Antibody Cocktail to each well.

14. Cover the plate with a foil plate sealer.

15. Incubate the plate at room temperature for 1 h at 800 rpm on an orbital shaker.

16. Repeat steps 6 to 10 (wash steps).

17. Prepare diluted Streptavidin-PE. Gently vortex the vial to mix.

1. 220 uL Streptavidin-PE concentrate + 5.35 mL wash buffer
2. Mix gently.
3. Cover with tin foil to protect from light.

18. Transfer the diluted Streptavidin-PE to a new pipette basin and cover with tin foil when not in use.

19. Using a multichannel pipette, add 50 uL of diluted Streptavidin-PE to each well.

20. Cover the plate with a foil plate sealer.

21. Incubate the plate at room temperature for 30 min at 800 rpm on an orbital shaker.

22. Repeat steps 6 to 10 (wash steps).

23. Add 100 uL of wash buffer to each well. Cover the plate with a foil plate sealer.

24. Incubate the plate at room temperature for 2 min at 800 rpm on an orbital shaker.

25. Read the plate using the Bioplex 200.

*Setting up Bioplex 200 for Assays*

1. Prepare assay protocols in software before beginning the assay.

2. Ensure the instrument has been validated recently. If not, validate the instrument using the MCV plate and Validation kit (Bio-Rad, Mississauga, ON, Cat # 171203001)

3. On the day of the assay:

1. Warm up the instrument during the 2- or 1-hour incubation. Instrument warmup takes approximately 30 min.
2. Calibrate the instrument before the assays using the MCV plate and calibration standards (Bio-Rad, Mississauga, ON; Cat #171203060

4. After calibration has been completed, open the appropriate protocol and run it to begin reading the plate. Be sure the following parameters on the software are inputted correctly.

1. 50 beads/region
2. Appropriate bead region for the analyte (refer to Certificate of Analysis; this should be done when setting up the protocol).

5. Export all data to Excel for analysis.

6. After the plate is read, shut down the instrument. Shut down takes approximately 10 min.

HUMAN MAGNETIC LUMINEX ASSAY – 6 PLEX CUSTOM ASSAY

Materials

- Pipettors (single and multi-channel)
- Pipette tips
- Orbital shaker
- Microcentrifuge
- Microcentrifuge tubes, 1.5 mL
- Handheld Magnetic Plate Washer (Thermo Fisher, Mississauga, ON)
- Custom magnetic Luminex Assay Kit (R&D Systems, Cat #: LXSAHM)
- Bioplex 200 (Bio-Rad, Mississauga, ON)
- Bioplex Manager Software (Bio-Rad, Mississauga, ON)

Method

1. Take Magnetic Luminex Assay kit out of the fridge and allow to come to room temperature.
2. Take samples out of the freezer and allow to thaw.

*Preparation of Samples*

1. Spin samples down at 16,000*g* for 4 min.
2. Dilute the samples 1:2 (as per the Certificate of Analysis)
3. 75 uL of serum sample in 75 uL Calibrator Diluent RD6-52

*Preparation of Standards*

1. Reconstitute one vial of each of the provided Human Standard cocktails provided (refer to Certificate of Analysis)

1. Standard K with 200 uL of Calibrator Diluent RD6-52
2. Standard B with 275 uL of Calibrator Diluent RD6-52
3. Standard A with 250 uL of Calibrator Diluent RD6-52
4. Standard F with 250 uL of Calibrator Diluent RD6-52

2. Allow the standard to sit with gentle shaking for at least 15 min.

3. Add 100 uL of Standard R solution to a tube containing 900 uL of Calibrator Diluent RD6-52. This is Standard 1.

4. To five 1.5 mL microcentrifuge tubes add 200 uL of Calibrator Diluent RD6-52. Label these tubes as Standards 2 to 6.

5. Mix Standard 1 by pipetting up and down three times.

6. Transfer 100 uL of Standard 1 into the tube for Standard 2 containing 200 uL of Calibrator Diluent RD6-52.

7. Mix Standard 2 thoroughly by pipetting up and down three times.

8. Transfer 100 uL of Standard 2 to the tube for Standard 3 containing 200 uL of Calibrator Diluent RD6-52.

9. Continue this procedure until you get to Standard 6. Do not transfer any material from the Standard 6 tube (the volume of this tube should be 300 uL)

10. Calibrator Diluent RD6-52 will serve as the blank for the assay.

*Preparation of Microparticle Cocktail*

1. Briefly centrifuge the microparticle cocktail vial for 30 s at 1000*g*.
2. Resuspend the particles by gentle vortexing the vial. Do not invert the vial!
3. Dilute the microparticle cocktail using Diluent RD2-1 in the mixing bottle provided.
4. 500 uL of microparticle cocktail + 5.00 mL of Diluent RD2-1
5. Be sure to keep the microparticles protected from light during handling.

*Assay Procedure*

1. To the 96 well plate, add 50 uL of standard or sample to the appropriate wells. Samples and standards are assayed in duplicate.

2. Resuspend the diluted microparticle cocktail by gently mixing the bottle. Transfer the cocktail to

a pipette basin and cover with tinfoil to protect from light.

3. Using a multichannel pipette, add 50 uL of microparticle cocktail to each well. Be sure to mix contents of the pipette basin regularly by gently rocking back and forth so particles do not settle to the bottom of the basin.

4. Cover the plate with a foil plate sealer.

5. Incubate the plate at room temperature for 2 h at 800 rpm using an orbital shaker.

6. Remove the plate from the orbital shaker and place it on the handheld magnetic plate washer. Ensure the plate is secure before beginning any wash steps.

7. Allow the plate to sit for 1 min so the microparticles can settle to the bottom of the well.

8. After 1 min, remove the liquid quickly by inverting the plate and magnetic plate washer over the sink.

9. Add 100 uL of wash buffer to each well. Allow to sit for 1 min. Cover the plate with tin foil between each wash.

10. After 1 min, remove the liquid from the plate. Repeat the addition of wash buffer (step 9) to the plate. Repeat the wash procedure for a total of three times.

11. Prepare diluted Biotin Antibody cocktail. Gently vortex the vial to mix.

1. 500 uL of concentrated Biotin Antibody + 5.00 mL of Diluent RD2-1
2. Mix gently.

12. Transfer the diluted Biotin Antibody cocktail to a new pipette basin.

13. Using a multichannel pipette, add 50 uL of diluted Biotin Antibody Cocktail to each well.

14. Cover the plate with a foil plate sealer.

15. Incubate the plate at room temperature for 1 h at 800 rpm on an orbital shaker.

16. Repeat steps 6 to 10 (wash steps).

17. Prepare diluted Streptavidin-PE. Gently vortex the vial to mix.

1. 220 uL Streptavidin-PE concentrate + 5.35 mL wash buffer
2. Mix gently.
3. Cover with tin foil to protect from light.

18. Transfer the diluted Streptavidin-PE to a new pipette basin and cover with tin foil when not in use.

19. Using a multichannel pipette, add 50 uL of diluted Streptavidin-PE to each well.

20. Cover the plate with a foil plate sealer.

21. Incubate the plate at room temperature for 30 min at 800 rpm on an orbital shaker.

22. Repeat steps 6 to 10 (wash steps).

23. Add 100 uL of wash buffer to each well. Cover the plate with a foil plate sealer.

24. Incubate the plate at room temperature for 2 min at 800 rpm on an orbital shaker.

25. Read the plate using the Bioplex 200.

*Setting up Bioplex 200 for Assays*

1. Prepare assay protocols in software before beginning the assay.

2. Ensure the instrument has been validated recently. If not, validate the instrument using the MCV plate and Validation kit (Bio-Rad, Mississauga, ON, Cat # 171203001)

3. On the day of the assay:

1. Warm up the instrument during the 2- or 1-h incubation. Instrument warmup takes approximately 30 min.
2. Calibrate the instrument before the assays using the MCV plate and calibration standards (Bio-Rad, Mississauga, ON; Cat #171203060)

4. After calibration has been completed, open the appropriate protocol and run it to begin reading the plate. Be sure the following parameters on the software are inputted correctly.

1. 50 beads/region
2. Appropriate bead region for the analyte (refer to Certificate of Analysis; this should be done when setting up the protocol).

5. Export all the data to Excel for analysis.

6. After the plate is read shut down the instrument. Shut down takes approximately 10 min.
